# EVI1 carboxy-terminal phosphorylation is ATM-mediated and sustains transcriptional modulation and self-renewal via enhanced CtBP1 association

**DOI:** 10.1093/nar/gky536

**Published:** 2018-06-25

**Authors:** Roberto Paredes, Marion Schneider, Adam Stevens, Daniel J White, Andrew J K Williamson, Joanne Muter, Stella Pearson, James R Kelly, Kathleen Connors, Daniel H Wiseman, John A Chadwick, Harald Löffler, Hsiang Ying Teng, Simon Lovell, Richard Unwin, Henri J van de Vrugt, Helen Smith, Olga Kustikova, Axel Schambach, Tim C P Somervaille, Andrew Pierce, Anthony D Whetton, Stefan Meyer

**Affiliations:** 1 Stem Cell and Leukaemia Proteomics Laboratory, Division of Cancer Sciences, Faculty of Biology, Medicine and Health, University of Manchester, Palatine Road, Manchester M20 3LI, UK; 2 Manchester Academic Health Science Centre, Manchester, UK; 3 Division of Developmental Biology and Medicine, Faculty of Biology, Medicine and Health M13 9WL, University of Manchester, UK; 4 Leukaemia Biology Group, CRUK Manchester Institute, Manchester M20 4XB, UK; 5 Clinical Cooperation Unit Molecular Hematology/Oncology, German Cancer Research Center (DKFZ) and Department of Internal Medicine V, University of Heidelberg, Heidelberg, Germany; 6 Evolution, Systems and Genomics Domain,Faculty of Biology, Medicine and Health, University of Manchester, Manchester M13 9PT, UK; 7 Oncogenetics, Department of Clinical Genetics, VU University Medical Center, Amsterdam, The Netherlands; 8 Institute of Experimental Hematology, Hannover Medical School; Hannover, Germany; 9 Stoller Biomarker Discovery Centre, University of Manchester, Manchester M13 9NQ, UK; 10 Department of Paediatric Haematology and Oncology, Royal Manchester Children's Hospital, Manchester M13 9WL, UK; 11 Young Oncology Unit, The Christie NHS Foundation Trust, Manchester M20 4XB, UK

## Abstract

The transcriptional regulator EVI1 has an essential role in early hematopoiesis and development. However, aberrantly high expression of *EVI1* has potent oncogenic properties and confers poor prognosis and chemo-resistance in leukemia and solid tumors. To investigate to what extent EVI1 function might be regulated by post-translational modifications we carried out mass spectrometry- and antibody-based analyses and uncovered an ATM-mediated double phosphorylation of EVI1 at the carboxy-terminal S858/S860 SQS motif. In the presence of genotoxic stress EVI1-WT (SQS), but not site mutated EVI1-AQA was able to maintain transcriptional patterns and transformation potency, while under standard conditions carboxy-terminal mutation had no effect. Maintenance of hematopoietic progenitor cell clonogenic potential was profoundly impaired with EVI1-AQA compared with EVI1-WT, in particular in the presence of genotoxic stress. Exploring mechanistic events underlying these observations, we showed that after genotoxic stress EVI1-WT, but not EVI1-AQA increased its level of association with its functionally essential interaction partner CtBP1, implying a role for ATM in regulating EVI1 protein interactions via phosphorylation. This aspect of EVI1 regulation is therapeutically relevant, as chemotherapy-induced genotoxicity might detrimentally sustain EVI1 function via stress response mediated phosphorylation, and ATM-inhibition might be of specific targeted benefit in EVI1-overexpressing malignancies.

## INTRODUCTION

EVI1 is a transcriptional regulator with an essential role in early development and hematopoiesis (1–3). However, aberrantly high expression of *EVI1*, which frequently results from chromosomal aberrations involving MECOM (MDS-EVI1 complex) locus on 3q26, where *EVI1* is encoded, has potent oncogenic properties with transformation capabilities *in vivo* and *in vitro* (4,5). In acute myeloid leukemia (AML) high *EVI1* expression is associated with poor response to cytotoxic treatment and adverse outcome (6,7). *EVI1* overexpression has been linked to leukemic transformation in children undergoing gene therapy (8), and individuals affected by Fanconi Anaemia (FA), which is an inherited DNA damage response defect with cancer predisposition (9,10). High *EVI1* expression conferring resistance to cytotoxic treatment and poor prognosis is also seen in other malignancies (11–14). Modulation of transcription by EVI1 is not understood in detail, and might, as for other transcriptional regulators, be partly dependent on the cell context of the *EVI1* overexpression event (15,16). Thus, *EVI1*-coregulated genes are variable in different types of leukemia (17), but nonetheless transcriptional patterns mediated by EVI1 in AML are enriched for genes involved in metabolic processes, differentiation and proliferation, and affect metabolic dependence on mitochondrial creatine kinase (18,19). Several transcripts are encoded in the MECOM locus, of which the main transcript encodes the 145 kDa EVI1 nuclear protein, which consists of an N-terminal zinc finger domain with seven motifs, a proline rich central repressor domain, a smaller C-terminal zinc finger domain with three motifs, and a carboxyterminal acidic domain. EVI1 modulates transcription by direct interaction of its zinc fingers with specific DNA sequences (20,21), and also via recruitment of other transcriptional regulators and chromatin modelling factors (22–25), which promotes the concept of EVI1 as a mediator of epigenetic changes that maintain a stem cell like phenotype with transforming capability (3). Specifically, the interaction of EVI1 with the co-repressor C-terminal binding protein 1 (CtBP1) has been shown to be essential for EVI1-mediated transformation (26,27). As post-translational modifications of EVI1 have been implicated in modulation of transforming ability and EVI1 protein interactions (28,29), we further investigated the role of phosphorylation in modulating EVI1 function. We report here on carboxy-terminal phosphorylation in response to genotoxic stress and associated consequences for EVI1 function. These define an opportunity for ameliorating treatment of diseases associated with EVI1 overexpression by targeting ATM kinase.

## MATERIALS AND METHODS

### Cell lines and tissue culture

The FA-derived AML cell line SB1690CB, which expresses high levels of EVI1, and the EVI1 negative AML cell line OCI-AML5 were maintained as described previously (10). HEK293T cells and Rat1 fibroblasts were used as previously described (28). Genotoxic stress was induced by treatment with H_2_O_2_ at concentrations previously used (30), or irradiation using a 320 kV X-ray system (Gulmay Medical Ltd) for suspension culture leukemia cells or using the image-guided micro-irradiation system for 96 well plates provided via the SARRP research platform (Xstrahl). Induction of activation of the DNA damage response was confirmed by demonstration of increased quantities of γH2AX foci and p53 phosphorylation. ATM inhibition was achieved with pretreatment for 1 h in the presence of 10 μM of the ATM-inhibitor KU55933 (Torcis).

### Antibodies

For detection of human EVI1 a polyclonal antibody raised against the N-terminal EVI1 epitope MKSEDYPHETMAPDI (Eurogentec), and the EVI1 antibodies #2265 and #2593 (Cell Signaling Technology, CST) were used. For detection of phosphorylated EVI1 we used phospho-specific, affinity purified rabbit polyclonal antibodies raised against EVI1-phospho-peptides NSNHGSQpSPRNVEC for serine 860 (S860) phosphorylation (anti-pS860-EVI1), and NSNHGpSQpSPRNVEC for serine 858 and serine 860 (S858/S860) double phosphorylation (anti-pS858/pS860-EVI1) (Eurogentec) (further details Supplementary Figure S1). For other antibodies see Supplementary Material Table S1.

### Immunoprecipitation, co-immunoprecipitation and western Blot

Cell lysis and immunoprecipitation were carried out as previously described (28). EVI1 was immunoprecipitated with EVI1 antibody #2593 (Cell Signaling) and captured with protein A sepharose beads. For immunoprecipitation of flag-tagged proteins transfected cells were incubated for one hour with FlagM2 magnetic beads (Sigma). Protein electrophoresis and western blots were carried out using standard methodologies (28). For quantitation of non-saturated western blot signals see Supplementary Methods and Supplementary Figure S2.

### Mass spectrometry

Immunoprecipitated EVI1 from 6 × 10^8^ SB1690CB AML cells was analyzed using multiple reaction monitoring-initiated detection and sequencing (MIDAS) (31). Following gel electrophoresis, the EVI1 containing band was excised and digested with trypsin. Peptides were separated by liquid chromatography prior to MIDAS using electrospray mass spectrometry on a 4000 Q-TRAP mass spectrometer (AB Sciex). MRM transitions were designed to detect 7–24 amino acid EVI1 peptides with serine, threonine or tyrosine phosphorylation within a Q1 *m/z* range from 400 to 1300 and in both a double and triple charge state. A Q3 mass of either 216.0 Da or Q1 minus 98 Da was used to identify tyrosine or serine/threonine phosphorylation, respectively. MS/MS data were interrogated using MASCOT and confirmed by manual inspection of spectra.

### Plasmids and site directed mutagenesis

The human EVI1 coding region was excised from pBABE-puro-flag-EVI1 (gift from Aubrey Thompson) (32) using SalI and EcoRI restriction sites and inserted into the SalI and EcoRI sites of pCMV-flag-5a. Substitution of S858 and S860 for alanine (A) to create the vector pCMV-EVI1-AQA-flag was done by site-directed mutagenesis using the QuikChange^®^ II XL Kit (Agilent). The Codon-optimized mouse Evi1 lentiviral vector pRRL.PPT.SF.EVI1mCo.IRES_EGFP.pre (19,33,34) was mutated as above to generate pRRL.PPT.SF.EVI1mCoAQA.IRES_EGFP.pre. Control pRRL.PPT.SF.IRES_EGFP.pre was generated by excision of the EVI1-ORF from pRRL.PPT.SF.EVI1mCo.IRES_EGFP.pre vector with BamHI restriction enzyme and re-ligated to create an empty backbone vector. Lentiviral packaging vectors pHCMV-G, pMDLg/pRRE and pRSV-Rev were used as described (35). Primer sequences are provided in the Supplementary Table S3. Confirmation of mutated sequence is illustrated in Supplementary Figure S3.

### Gene expression analysis

Reporter gene assays were carried out in HEK293T cells as described before (details in Supplementary Material) (28), with RT-PCR monitored *EVI1-*expression (Supplementary Figure S4A). For Poly-A RNA sequencing (RNA*seq*) analysis of EVI1-mediated modulation of gene expression RNA was extracted from HEK293 cells, which were subjected to transient transfection using half confluent cultures with pCMV-flag, pCMV-EVI1-WT-flag or pCMV-EVI1-AQA-flag, exposed to 150 μM H_2_O_2_ or left untreated for 8 h. Equal levels of EVI1 levels were monitored by western blot (Supplementary Figure S4B). Libraries were prepared with the Lexogen QuantSeq 3′ mRNA-Seq Library Prep Kit for Illumina (FWD) using an input of 200 ng and performing 14 cycles of amplification. Indexed libraries were then quantified using the Kapa Illumina Library quantification kit (Cat. 07960336001) and pooled. 1 × 75 bp sequence reads were generated by clustering 2.0 pM of the library pool on a NextSeq500 High throughput run. Ordered BAM files were generated against the human genome feature file Homo_sapiens.GRCh38.90.gtf downloaded from Ensembl (ftp://ftp.ensembl.org/pub/current_gtf) (36). Data analysis was performed using Qlucore Omics Explorer 3.3 (Qlucore, Lund, Sweden) with a FPKM (Fragments Per Kilobase Million) cut-off of 10. To identify significant differentially regulated transcripts, RNAseq expression data analysis was carried out by applying a group ANOVA with *P* < 0.01 to the entire dataset. For illustration of the comparisons between the effect on transcription with EVI1-WT and EVI1-AQA in relation to untransfected cells and empty vector-transfected control cells with and without DNA damage, values were normalized to the mean of 0 and a variance of one. In addition, differences between expression levels at individual conditions were assessed by two group comparison (*t*-test). Biological pathways associated with differential gene expression were determined using a right-sided Fisher's exact test (Ingenuity Pathways Analysis, Qiagen). The effect on levels of expression detected by RNA*seq* were confirmed by RT-PCR for selected transcripts using *taqman* technology with housekeeper transcripts (*YWHAZ* and *β-ACTIN)* chosen from a tested pool of housekeeper genes for minimal variation between samples and conditions (primer sequences and detailed methodology in supplementary material). RT-PCR data was processed for ΔΔ–CT calculation normalized to housekeeping transcripts.

### Rat-1 fibroblast transformation assay

Retroviral transduction of Rat-1 fibroblasts with human EVI1 was carried out as described previously (28), using FUGENHD (Promega)- transfected packaging Plat-E cells (MSCV-EVI1-IRES-GFP, MSCV-EVI1-AQA-IRES-GFP or empty vector control MSCV-IRES-GFP). After 4 days cells were FACS-sorted by GFP, and equal levels of EVI1 expression were confirmed by western blot (Supplementary Figure S5A–C). GFP^+^ cells (10^4^) were seeded in untreated methylcellulose medium (MethoCult™ M3231, Stem Cell Technology), or supplemented with 30 μM of H_2_O_2_. Alternatively, 1 × 10^5^ cells in 100 μl (96-well plate) were left untreated or irradiated (0.5 or 2 Gy). After 14 days, colony number and size were quantified and documented using a DMIL inverted microscope fitted with a MC170 HD camera (Leica) (Supplementary Figure S5D). Induction of DNA damage was assessed by induction of γH2AX foci (Supplementary Figure S5E).

### Serial replating of hematopoietic progenitors

Hematopoietic c-Kit^+^ progenitor cells were isolated from bone marrow of 8–10 week old C57/BL6 mice as previously described (28,37). Lentiviral mediated transduction was carried out with EVI1 vectors pRRL.PPT.SF.EVI1mCo.IRES_EGFP.pre, and site mutated pRRL.PPT.SF.EVI1mCoAQA.IRES_EGFP.pre as described (19,38). In brief, cells cultured in medium supplemented with 4 μg/ml protamine (Sigma) were sequentially infected with two viral batches by spinoculation (60 min at 1250 × *g* at 32°C), and cultured for two days in pre-stimulation serum free cytokine supplemented medium (StemCell Technologies), prior to FACS selection of GFP^+^ cells. Equal transduction efficiency for EVI1-WT and EVI1-AQA was monitored by RT-PCR and quantitation of fluorescent signal (Supplementary Figures S6A and B). For replating 2 × 10^4^ GFP^+^ cells were plated in cytokine supplemented Methocult M3231 medium, without and with H_2_O_2_ at the prior demined IC_50_ dose (30 μM), with induction of genotoxic stress monitored by the induction of γH2AX foci (Supplementary Figure S6C and D). After 7 days in culture, colonies were counted and morphologically assessed (Supplementary Figure S6E). Then cells were harvested, and 2 × 10^4^ cells were replated as before. Colonies were scored for subsequent rounds of replating as above, and previously H_2_O_2_ treated colonies were split and either treated again or left untreated. For morphological assessment cytospin preparations were stained with May-Grünwald Giemsa (Supplementary Figure S6F), and 200 cells per preparation were assessed.

### Immunofluorescence (IF)

For immunofluorescence cells were incubated with antibodies following standard procedures and image generation. Spatial association of EVI1 and CtBP1 was quantified by determining linear signal correlation by Pearson product-moment correlation coefficient (39) (further details in Supplementary Methods). Induction of the DNA damage response was confirmed by demonstration of increased frequency of γH2AX foci (Supplementary Figure S7)

## RESULTS

### DNA damage mediates sequential phosphorylation of EVI1 on the carboxy-terminal SQS motif

**Figure 1.**
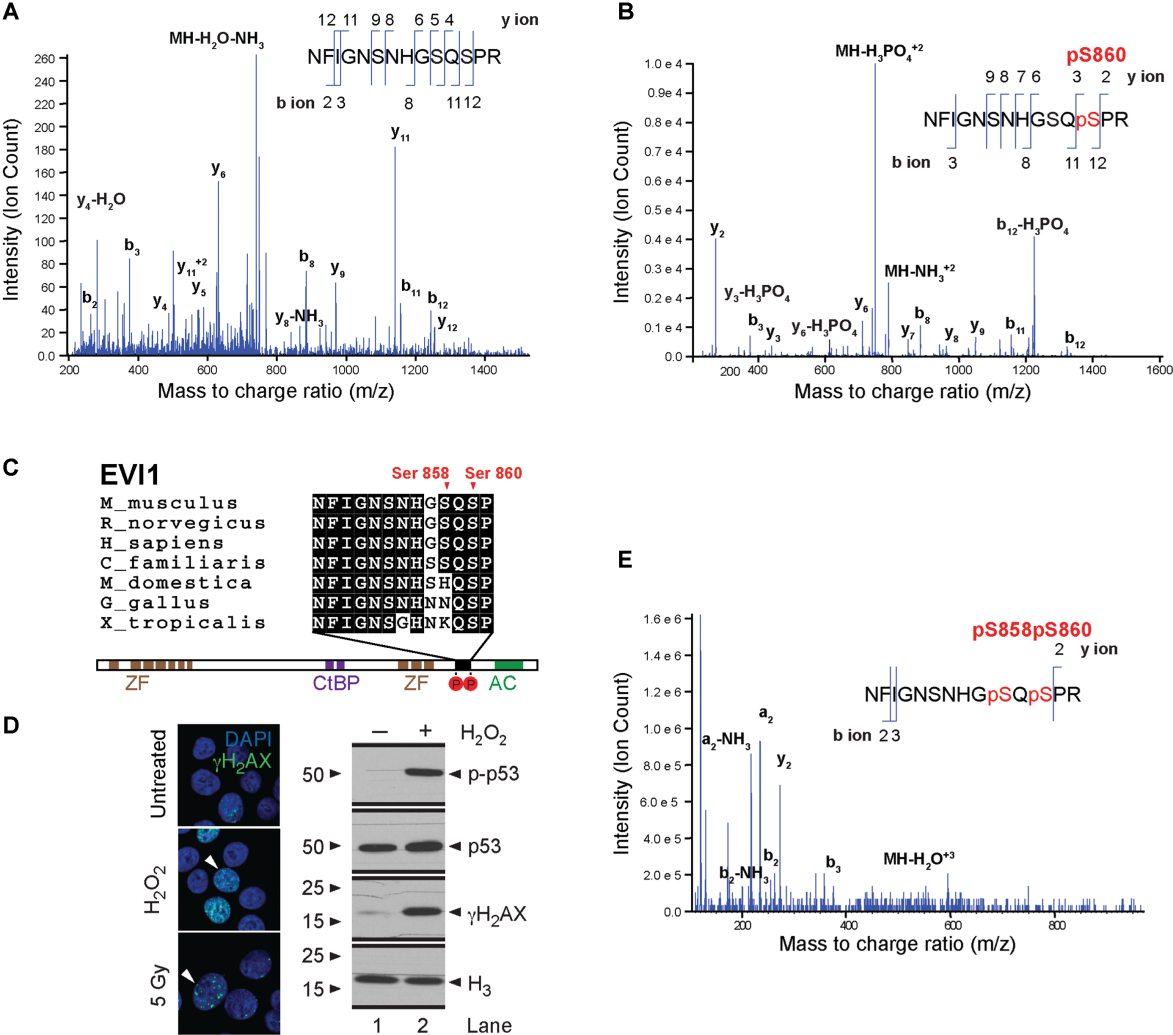
*Carboxy-terminal EVI1 phosphorylation*. Mass spectrometry analysis of the EVI1 peptide Asn849-Arg862 from SB1690CB AML cells, demonstrating the presence of non-phosphorylated (**A**) and single Ser860 phosphorylated peptides (**B**) in untreated SB1690CB cells. y- and b-type ions illustrating their position within the peptide sequence indicated. The mass/charge ratio (m/z) of the precursor ion is also shown. (**C**) Schematic representation of the EVI1 protein with sequence alignment of EVI1 from other species as indicated, showing DNA binding zinc finger domains (ZF), CtBP1 binding motifs (CtBP) and acidic domain (AC). Carboxy-terminal phosphorylation sites are shown in red circles and annotated in relation to the EVI1-SQS containing epitope alignment. (**D**) Confirmation of induction of DNA damage by γH2AX foci formation after H_2_O_2_ or radiation treatment of SB1690CB cells (left panel). White arrowheads denote cells with increased number of foci (green signal). DAPI co-stain for the nucleus (blue signal). Western blot (right panel) of *γ*H2AX and p-p53 (Ser15) as markers of DNA damage response activation and ATM activity after H_2_O_2_-treatment of SB1690CB cells. (**E**) EVI1 MS spectrum obtained for peptide Asn849-Arg862 from irradiated SB1690CB cells, inferring double phosphorylation of the carboxy-terminal S858/S860 SQS motif.

**Figure 2.**
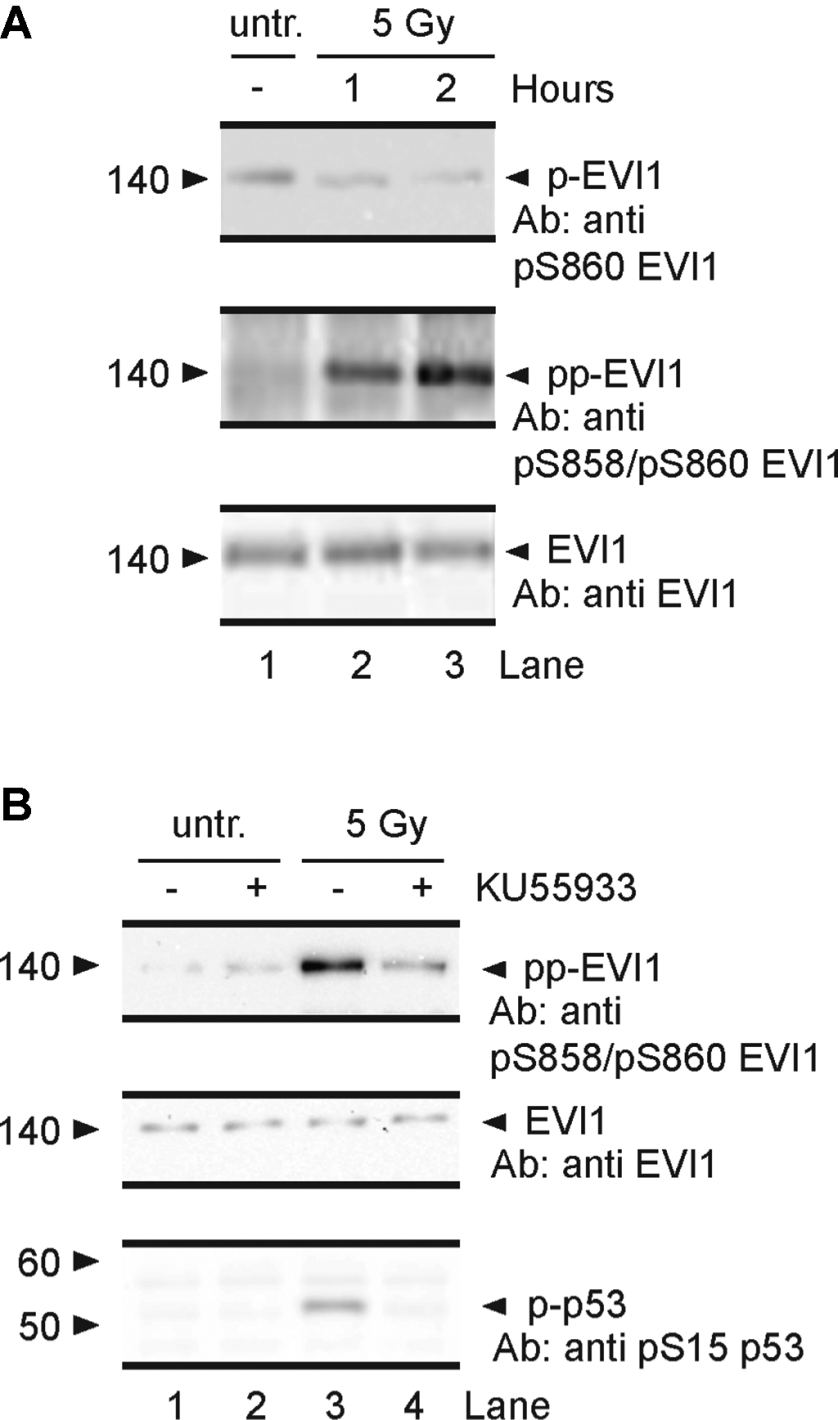
*Induction of EVI1 double phosphorylation by DNA damage*. (**A**) Immunoprecipitated EVI1 from SB1690CB cells probed with anti-pS860-EVI1 (upper panel), and anti-pS858/pS860-EVI1 antibody, untreated (lane 1), and after 1 and 2 h (lane 2 and 3) post radiation. (**B**) Western blot (WB) of EVI1-immunoprecipitates from untreated (lanes 1 and 2) and irradiated (lanes 3 and 4) SB1690CB cells pre-treated (1 h) with 10 μM ATM-kinase inhibitor KU55933 (lanes 2 and 4), and not pre-treated (lanes 1 and 3). Membranes were probed for doubly phosphorylated EVI1 with anti-pS858/pS860-EVI1 antibody. Middle panel: membrane as upper panel re-probed with pan-EVI1 antibody. Lower panel: WB of p-p53 (Ser15) carried out on input samples as positive control for ATM-activity and ATM-inhibition.

To identify phosphorylation events on EVI1, we carried out a selective reaction monitoring mass spectrometric analysis of endogenously expressed EVI1 immunoprecipitated from EVI1-overexpressing SB1690CB AML cells. Under standard conditions we identified the HFIGNSNHGSQSPR (aa 849–862) peptide (NCBI accession: NP_001098548.2) (Figure 1A). This peptide was also identified with a single site of phosphorylation (HFIGNSNHGSQpSPR), for which the MS/MS spectrum indicated serine phosphorylation at S860 (Figure 1B). This peptide involves an ATM (ataxia telangiectasia mutant)—kinase specific SQS motif (40), which is conserved across species (Figure 1C). We exposed cells to genotoxic stress by irradiation or H_2_O_2_, and confirmed induction of DNA damage by demonstration of increased γH2AX foci and p53 phosphorylation (Figure 1D). Mass spectrometric analysis of immunoprecipitated EVI1 after DNA damage uncovered a doubly phosphorylated EVI1-peptide at residues S858 and S860 (Figure 1E). Western blot analysis of EVI1 using antibodies specifically raised against EVI1 peptides phosphorylated only at S860, or S858 plus S860 respectively, confirmed a rapid induction of doubly phosphorylated EVI1 both at S858 and S860 together after irradiation or H_2_O_2_ treatment. This event was only detectable at very low levels in non-stressed cells, while correspondingly the signal for single phosphorylation at S860 decreased (Figure 2A). Pre-treatment with the ATM inhibitor KU55933 reduced the induction of the doubly phosphorylated (S858/S860) EVI1 after IR (Figure 2B). Mass spectrometric analysis of EVI1 repeatedly detected non-phosphorylated and EVI1 phosphorylated at S860 in untreated cells. After genotoxic stress EVI1 peptides with double phosphorylation at S858 and S860 were more abundant. However, a peptide indicating single phosphorylation on S858 alone was never detected. These findings suggest that the S858 phosphorylation is dynamically regulated in response to genotoxic stress after S860 phosphorylation.

### EVI1 transcriptional changes in the DNA damage response are sustained by carboxy-terminal SQS-phosphorylation

**Figure 3.**
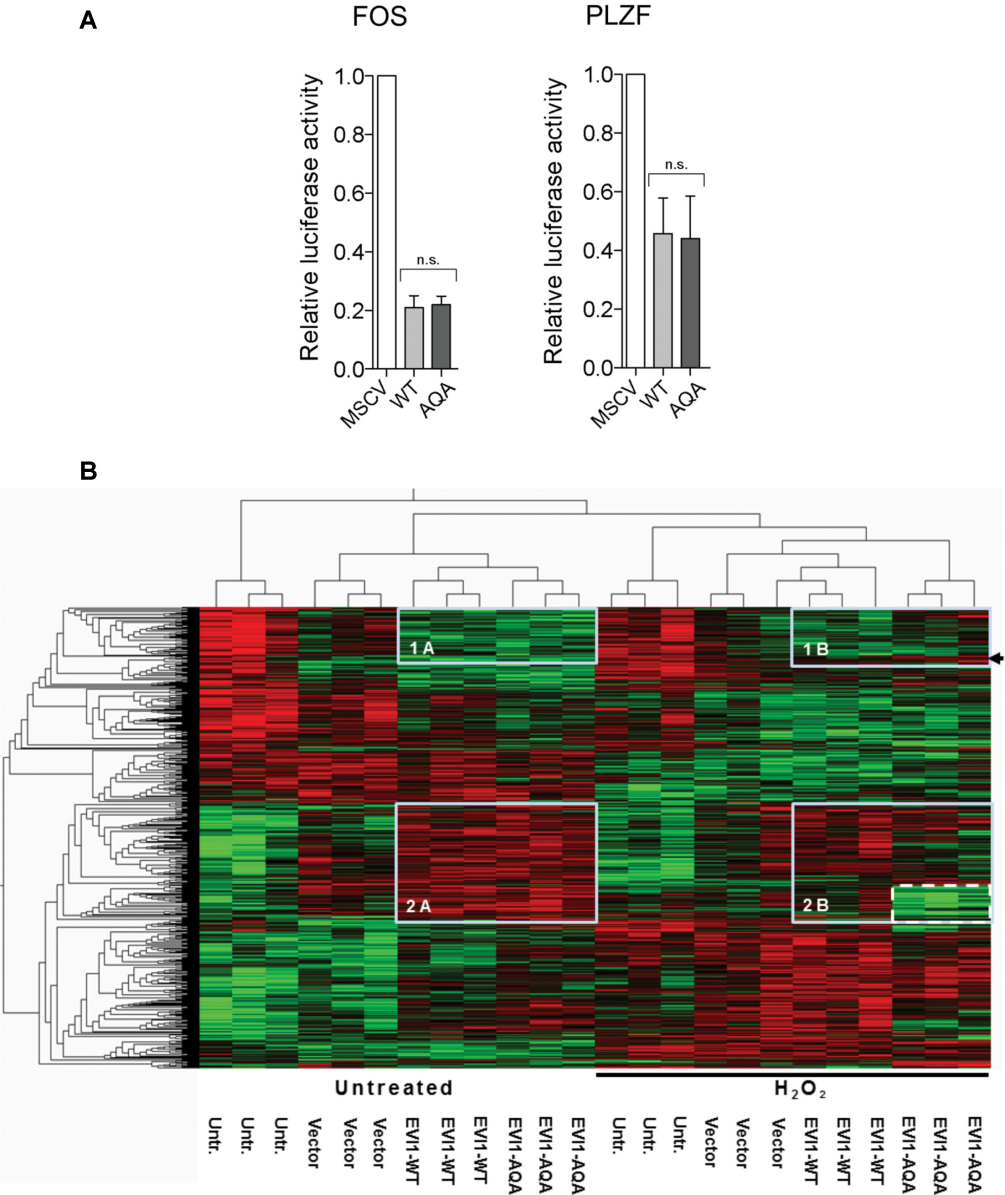
*Effect of EVI1-WT and EVI1-AQA on transcription*. (**A**) Luciferase assays on promoters of EVI1-repressed *PLZF* (left panel) and *FOS* (right panel) transcripts (*n* = 4, *t*-test, ns: not significant). (**B**) Heat map illustration of significantly differentially expressed transcripts (*P* < 0.01) after transfection with EVI1-WT and EVI1-AQA of untreated cells (left half of the heatmap), and after treatment with H_2_O_2_ (150 μM, right half of the heat map, indicated by black line) (*n* = 1306). The heatmap was generated applying a group ANOVA analysis to the entire dataset. Clusters of EVI1 regulated transcripts were identified by dendrogram delineation, and boxed for repressed (1A and 1B), and upregulated transcripts (2A and 2B). Patterns of sub-clusters in H_2_O_2_ treated cells with different changes comparing EVI-WT with EVI1-AQA indicated by arrow in 1B and dashed box in 2B.

To functionally assess the carboxy-terminal SQS phosphorylation we generated site mutated EVI1constructs to mimic non-phosphorylated, non-phosphorylatable EVI1 (EVI1-AQA), for comparison with the wild type EVI1-SQS (EVI1- WT) constructs. We first assessed target-gene promoter repression of EVI1-AQA compared with EVI1-WT and measured EVI1-mediated repression of *FOS* and *PLZF* (41,42). EVI1-WT and EVI1-AQA mutant repressed *PLZF* promoter, regulated by the EVI1 N-terminal zinc-finger motif, and C-terminal zinc-finger motif regulated *FOS* promoter equally, indicating that EVI1 promoter repression was not affected by mutation of S858 and S860 (Figure 3A). We next compared the effect on transcriptional patterns mediated by EVI1-WT and EVI1-AQA. An RNAs*eq* expression dataset was generated from untransfected, empty vector (vector-), EVI1-WT and EVI1-AQA transfected HEK293 cells, with and without DNA damage mediated by H_2_O_2_. We applied a group ANOVA with *P* < 0.01 to the entire dataset and identified 1306 significantly differentially expressed transcripts (Supplementary Table S4, Sheet 1). Further detailed analysis of untreated EVI1-WT and EVI1-AQA compared to vector only transfected cells by two group comparison showed a strong overlap (Supplementary Table S4, Sheet 2): Of 62 significantly (*P* < 0.05) upregulated transcripts with EVI1-WT transfection compared to empty vector, 22 are also significantly up-regulated in EVI1-AQA transfected cells. Likewise, of 90 significantly repressed transcripts with EVI1-WT transfection, 30 are also significantly repressed in EVI1-AQA transfected cells. However, in the presence of damage, 10 of 41 upregulated transcripts in EVI1-WT transfected cells were also significantly upregulated in EVI1-AQA transfected cells, and of 47 repressed transcripts in EVI1-WT transfected cells, only 7 were also significantly repressed by EVI1-AQA transfection. Pathways affected by EVI1-WT transfection in the presence of genotoxic stress in this model included fucose-carbohydrate biosynthesis, death-receptor- and MAPK signaling (Supplementary Table S4, excel file, sheet 3). A heat map of 1306 the differentially expressed transcripts identified by group ANOVA when EVI1-WT and EVI1-AQA were compared with untransfected cells and empty vector control in untreated cells and with H_2_O_2_ mediated DNA damage is clearly divided by vertical clustering in two halves by H_2_O_2_-induced genotoxic stress (Figure 3B). In untreated EVI1-transfected cells, horizontal dendrogram delineation separates two main clusters of EVI1-regulated transcripts, compared with non-transfected and vector-control transfected cells (boxed). In cluster 1, containing 139 transcripts, EVI1 mediated lower expression under normal conditions. For cluster 2, containing 328 transcripts, EVI1 mediated higher expression. Strikingly, when comparing heat-map patterns of untreated cells transfected with EVI1-WT with patterns from EVI1-AQA mutant transfected cells, the patterns boxed in cluster 1A and 2A are almost identical. Analysis in the same fashion of cells exposed to genotoxic stress by H_2_O_2_ showed that EVI1-WT mediated expression changes are clustering and largely overlapping with those delineated in untreated cells. However, in the presence of genotoxic stress the expression patterns mediated by EVI1-WT compared to those mediated by EVI1-AQA are more diverse. In the cluster 1B a distinct sub-cluster of 14 transcripts is delineated with decreased expression in untreated EVI1-WT and EVI1-AQA transfected cells, but in response to damage are not repressed in the non-phosphorylatable EVI1-AQA (Figure 3B, arrow). In cluster 2B there are multiple transcripts that are higher expressed in untreated and damaged EVI1-WT transfected cells, but not in damaged EVI1-AQA transfected cells, which form a distinct sub-cluster of 93 transcripts (Figure 3B, dashed box). The modulatory effect on transcriptional patterns mediated by carboxy-terminal mutated EVI1-AQA is stronger in the presence of genotoxic stress.

### EVI1 carboxy-terminal phosphorylation sustains EVI1 transforming ability in the DNA damage response

**Figure 4.**
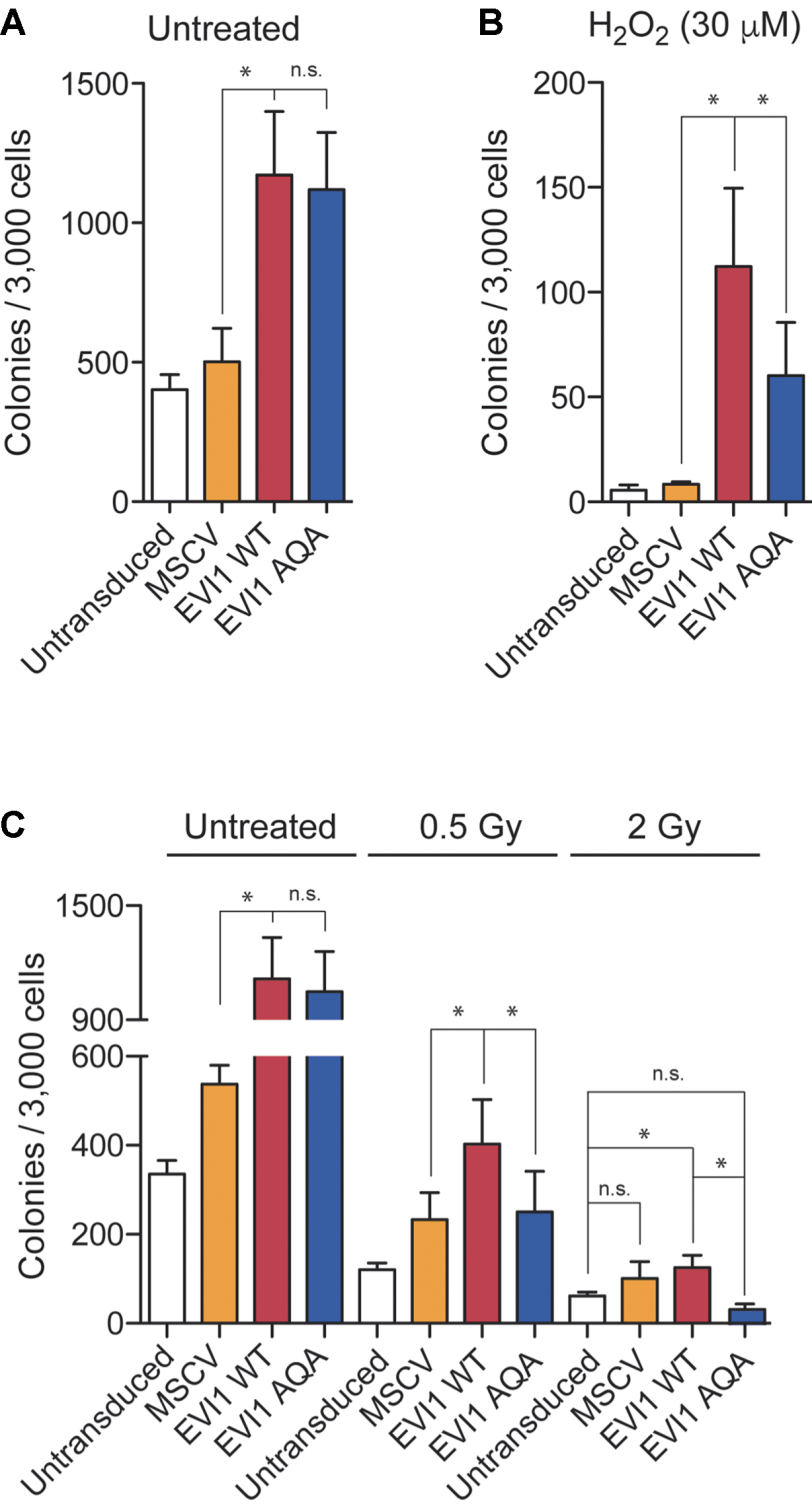
*EVI1-mediated Rat-1 fibroblast transformation*. (**A**) Rat-1 fibroblast colonies of untransduced, stably MSCV-, EVI1-WT- or EVI1-AQA-transduced fibroblasts. Rat-1 fibroblasts were left untreated, H_2_O_2_-treated (**B**) or irradiated with 0.5 and 2Gy as indicated (**C**) (*n* = 4). Statistical analysis: *t*-test (*P < 0.05, ns: not significant).

To investigate to what extent the double phosphorylation at S858 and S860 also sustains EVI1-mediated transformation, we compared the effect of EVI1-WT with the EVI1-AQA mutant on Rat-1 fibroblast transformation (5). Equal expression of EVI1-WT and EVI1-AQA constructs in Rat-1 fibroblast cultures and activation of the DNA damage response in transduced cells was confirmed (Supplementary Figure 5A–E). Transduction with EVI1-WT resulted in a significant 2.2-fold (mean, *n* = 4) increase in colony numbers after 14 days compared with vector only transduction, which under standard conditions was not significantly different from transduction with mutated EVI1-AQA (Figure 4A). However, in the presence of genotoxic stress mediated by H_2_O_2_, EVI1-WT confers a 10.1-fold increase in colony numbers compared to vector only, while EVI1-AQA had a markedly weaker effect (Figure 4B). Radiation had a more profound effect on Rat-1 transformation. At 0.5 Gy EVI1-WT conferred doubling of colony numbers, while EVI1-AQA transduction did not significantly increase colony numbers compared with vector only. At 2 Gy only EVI1-WT conferred any significant increase in colony numbers compared to untransduced cells, while no significant increase in colony numbers was seen with EVI1-AQA transfection (Figure 4C).

### The EVI1 carboxy-terminal SQS motif sustains hematopoietic self-renewal

**Figure 5.**
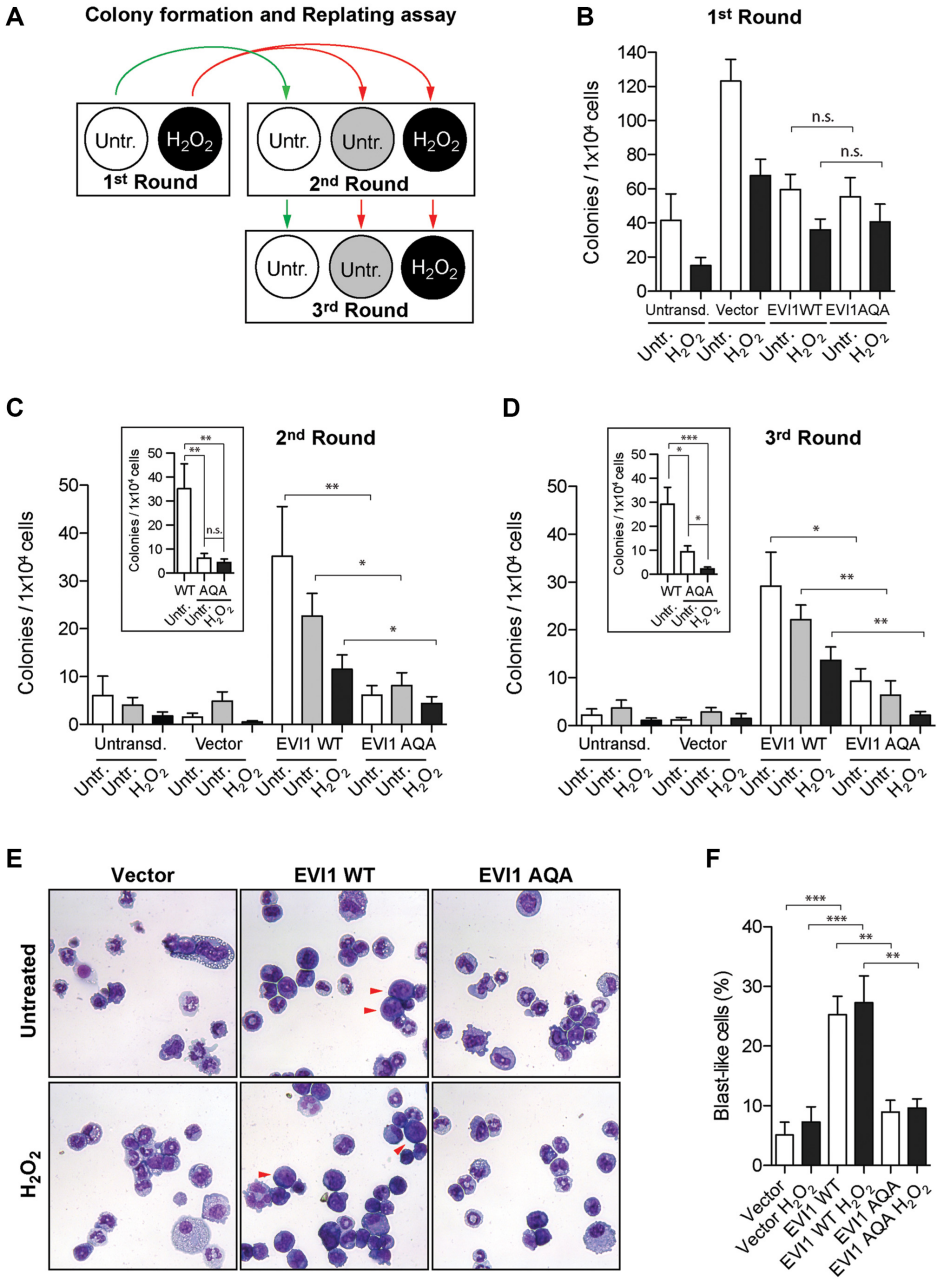
*EVI1-mediated serial replating of murine hematopoietic progenitor cells*. (**A**) Schematic representation of hematopoietic progenitor clonogenic replating: cKit+ cells were transduced, selected for transduction, and plated in the presence or absence of H_2_O_2_ (first round). Colonies were counted after 7 days, harvested and replated (second round). H_2_O_2_ treated cells were split in two batches (red arrows) to be left untreated or treated with a second dose of H_2_O_2_. Untreated cells were re-plated untreated (green arrow). Remaining cells were used for a third round of replating. (**B**) Colony count after first round (*n* = 8, from eight different mice, black: H_2_O_2_ treated). (**C** and **D**) Colony counts after the second and third rounds of replating (*n* = 8, from eight different mice). White: untreated; gray: untreated cells replated from previously H_2_O_2_ treated colonies; black: H_2_O_2_ treatment in all rounds. Inserts C and D: direct comparison between untreated EVI1-WT and EVI1-AQA transduction with and without damage. Statistical analysis: paired *t-*test (*P < 0.05, **P < 0.01). (**E**) May-Grünwald Giemsa stained cells from colonies after first culture. Red arrowheads point typical blast-like cell morphology. (**F**) Blast-like cells quantitation from the first round of replating. Statistical analysis: one-way ANOVA (**P < 0.01, ***P < 0.001).

Transduction with EVI1 has been shown to increase replating capacity of hematopoietic progenitor cells, reflecting the importance of EVI1 for hematopoietic self-renewal (3,19). As ATM-knockout leads to haematopoietic stem cell exhaustion (43), we surmised that the ATM-mediated phosphorylation event at S858/S860 would be linked to EVI1-mediated self-renewal. We confirmed the replating potency of EVI-WT with murine c-Kit^+^ hematopoietic progenitor cells for three rounds of replating, untreated and in the presence of genotoxic stress (Figure 5A) (*n* = 8, from eight mice). Transduction with EVI1-AQA did not affect replating efficiency compared to EVI1-WT in the first round of plating (Figure 5B); vector only transfected cells also formed colonies. However, when replated in the second and third round, when vector only transduced cells did not form colonies, colony forming capacity of EVI1-AQA was significantly reduced compared with EVI1-WT (Figure 5C and D), even more profoundly in the presence of genotoxic stress. In line with these observations and published data (19), morphological analysis of EVI1-WT transduced colonies revealed a significant proportion of immature myeloid cells with blast-like morphology. Comparing EVI-WT- with EVI1-AQA-transfected colonies uncovered a significantly reduced number of cells with blast-like morphology in EVI1-AQA transduced colonies even in the first round of replating (Figure 5E and F). These observations imply that maintenance of a hematopoietic blast-like and self-renewal phenotype by EVI1 is partly dependent on the phosphorylatable carboxy-terminal SQS motif.

### Increased spatial association of EVI1 with CtBP1 in response to genotoxic stress

**Figure 6.**
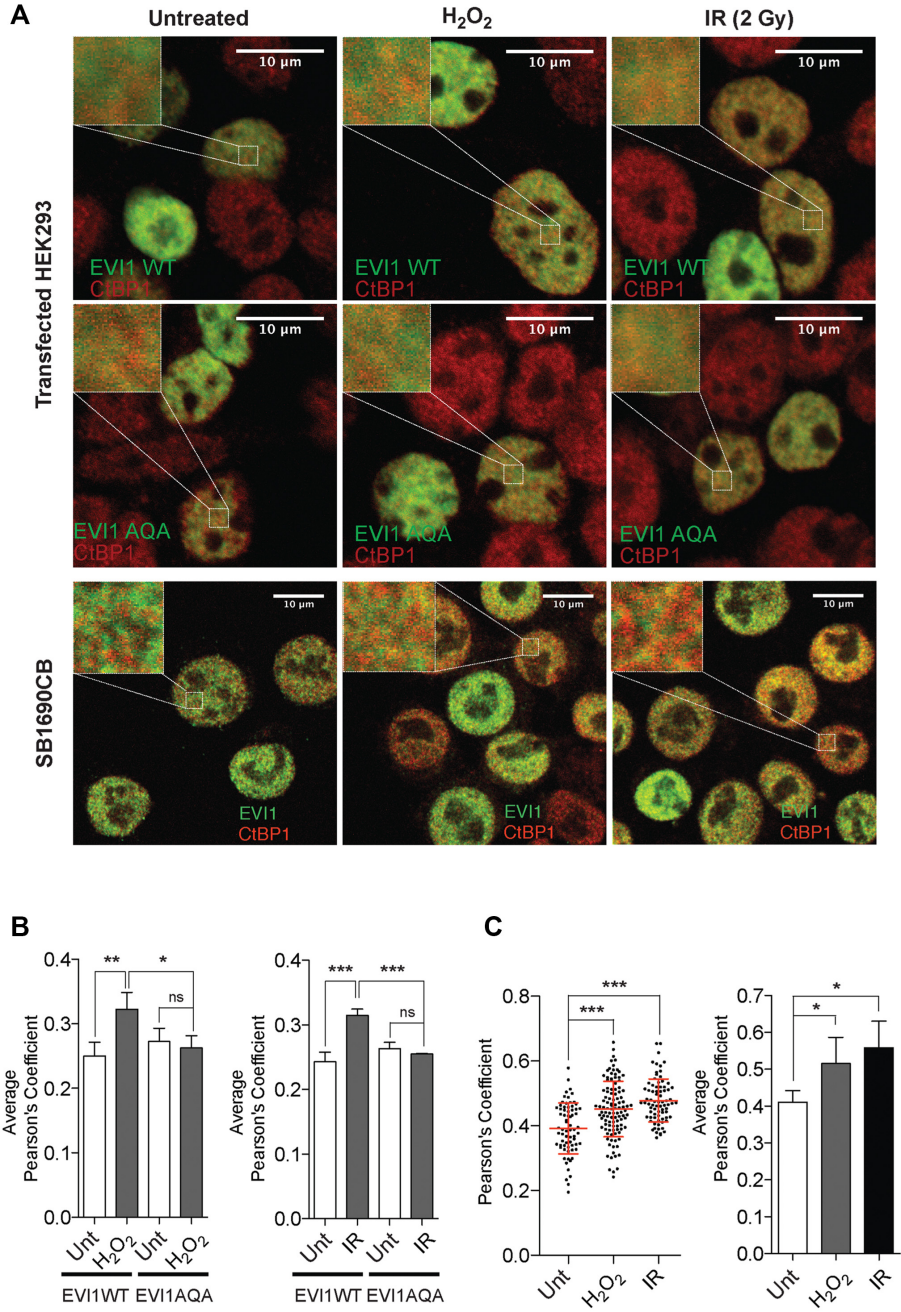
*Nuclear association of EVI1 with CtBP1*. (**A**) EVI1 (green) and CtBP1 (red) immunofluorescence signal of HEK293T cells transfected with EVI1-WT (upper panel) and EVI1-AQA mutant (middle panel), or SB1690CB AML cells (lower panel) after genotoxic stress induced either with H_2_O_2_ treatment or irradiation (2 Gy). Left top corner inserts represent 4 μm^2^ regions of interests (ROI) in HEK293T cells or 3 μm^2^ ROIs in SB1690CB cells (eight ROIs/cell analyzed). (**B**) Quantification of EVI1 and CtBP1 co-localization in EVI1-WT or EVI1-AQA transfected HEK293T cells after genotoxic stress. Degree of co-localization is expressed as Pearson's Coefficient (P’sC). Each bar represents the average P’sC of independent experiments (*n* = 3) for H_2_O_2_ (left panel) and irradiated cells (right panel). (**C**) Distribution of P’sC for endogenously expressed EVI1 and CtBP1 co-localization in SB1690CB leukemia cells after H_2_O_2_ treatment or irradiation. Each dot represents a single cell (left panel). Average P’sC of independent experiments (*n* = 3) plotted as bar graphs (right panel). Statistical analysis: one-way ANOVA (*P < 0.05, **P < 0.01, ***P < 0.001, ns = not significant).

To explain the distinct effect of DNA damage induced phosphorylation on EVI1 transcription and transforming ability we hypothesized that these effects would be mediated by alteration of important EVI1 interactions with other transcriptionally active proteins. This seemed in particular plausible as the carboxy-terminal region has not been implicated in direct interaction of EVI1 with DNA, and SQS to AQA motif mutation had no effect on promoter repression (see above). As the interaction with the co-repressor CtBP1 is essential for EVI1-mediated transformation (22,27), and CtBP1 itself has a role in the maintenance of genomic integrity and DNA damage response (44,45), we investigated the effect of EVI1 carboxy-terminal phosphorylation, which does not directly involve the CtBP1 binding sites of EVI1 (27), on the EVI1-interaction with CtBP1 in the presence of genotoxic stress monitored by induction of γH2AX foci (Supplementary Figure S7B and C). As the DNA damage response has been implicated in ATM-mediated phosphorylation determining cellular localization of proteins (40,46,47), we assessed the nuclear localization of EVI1 in relationship to CtBP1 with and without genotoxic stress. Under non-stressed conditions non-phosphorylatable mutant EVI1-AQA, which cannot be phosphorylated on this ATM consensus site, co-localized with CtBP1 in transfected HEK293T cells to the same degree as EVI1-WT. However, in the presence of genotoxic stress mediated by H_2_O_2_ or radiation, we observed a highly significant increase in spatial association of EVI1-WT with CtBP1 of 22.1% ±- 4.4 SEM with H_2_O_2_ (*n* = 4, *P* < 0.01), and of 22.7% ± 2.4 SEM after radiation (*P* < 0.001) (Figure 6A, upper two panels and 6B). Non-phosphorylatable EVI1-AQA mutant failed to increase proximity in association with CtBP1 after treatment with H_2_O_2_ or radiation. In endogenously EVI1-expressing SB1690CB AML cells, the highly significant increase in the degree of co-localization with CtBP1 in the presence of genotoxic stress was confirmed with cells with increases of 18.7% ± 3.9 SEM) after H_2_O_2_ treatment, and 24.9% ± 4.8 SEM after radiation (*n* = 3, *P* < 0.01) (Figure 6A, lower panel, and C).

### Increased EVI1-CtBP1 interaction after genotoxic stress

**Figure 7.**
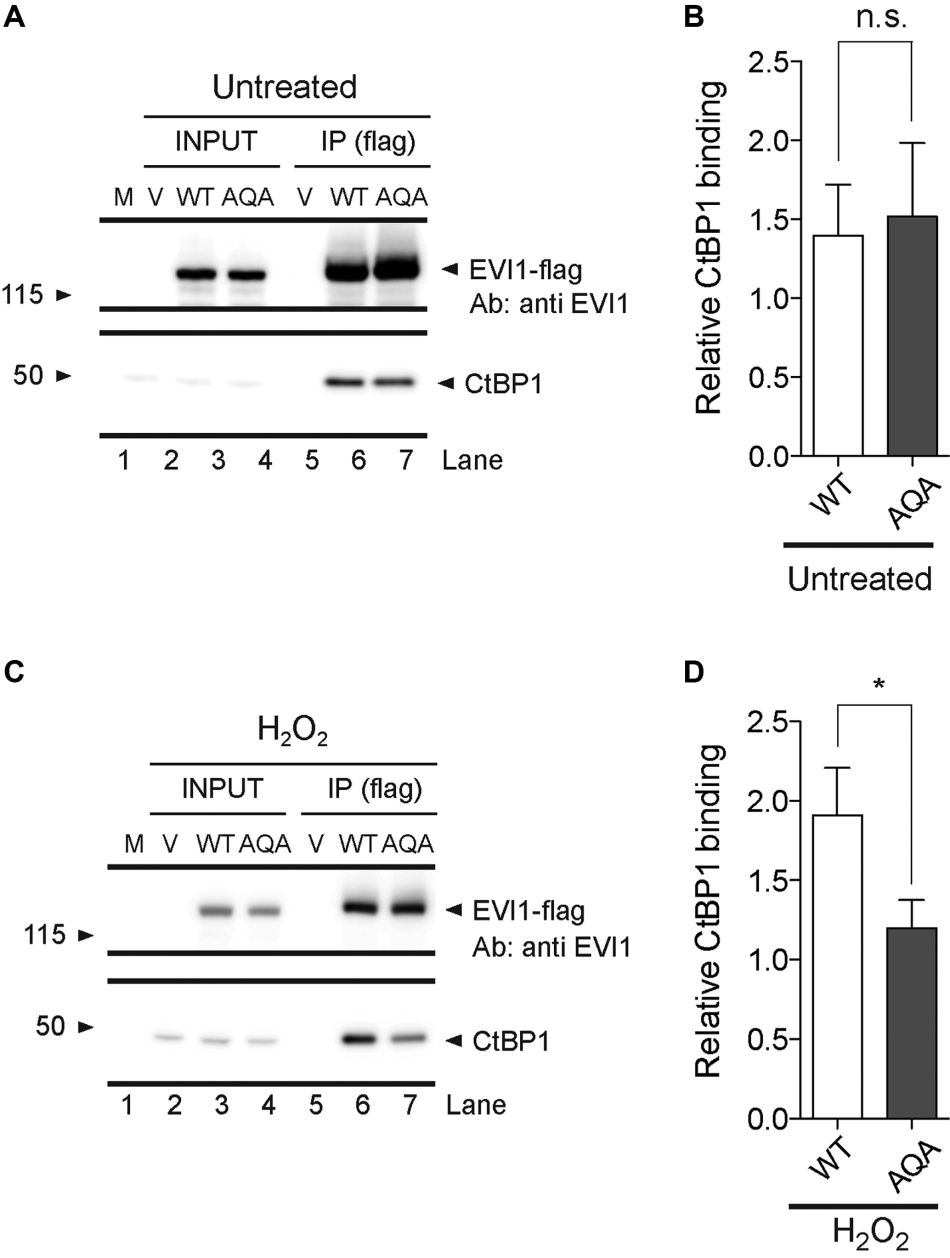
*Effect of genotoxic stress on EVI1-CtBP1 interaction*. (**A**) Western blot analysis of flag-antibody immunoprecipitated tagged EVI1 and co-immunoprecipitated CtBP1 from total cell protein extracts of untreated Flag-tagged EVI1-WT or EVI1-AQA transfected HEK293 cells (upper panel: EVI1; lower panel: CtBP1). (**B**) Signal quantification of serial biological replicates (*n* = 3) at 3 min (n.s. = not significant, *t*-test). (**C**) Western blot analysis of flag-antibody immunoprecipitated EVI1 and co-immunoprecipitated CtBP1 from total cell protein extracts of H_2_O_2_ treated Flag-tagged EVI1-WT and EVI1-AQA transfected cells (upper panel: EVI1; lower panel CtBP1). (**D**) Signal quantification of biological replicates (*n* = 3) (*P < 0.05, *t*-test).

In order to explore if the phosphorylation-mediated increase in co-localization of EVI1 with CtBP1 reflects EVI1 interaction, we performed co-immunoprecipitations using Flag-tagged EVI1-WT and EVI1-AQA in HEK293T cells. Comparing EVI1-WT with EVI1-AQA mutant, the levels of co-immunoprecipitated CtBP1 for EVI1-WT and EVI1-AQA mutant were not different under standard conditions (Figure 7A and B). However, the non-phosphorylatable EVI1-AQA mutant showed 36.9% ± 6.1 SEM less co-immunoprecipitated CtBP1 (*P* < 0.05) after treatment with H_2_O_2_ compared with EVI1-WT (Figure 7C and D), implying greater EVI1-CtBP1 affinity after damage-mediated carboxyterminal EVI1-phosphorylation. Thus, the CtBP1-EVI1 interaction in the DNA damage response is promoted by the carboxy-terminal EVI1 phosphorylation, and points to the DNA damage response affecting transcriptionally relevant EVI1 protein complex formations.

## DISCUSSION

EVI1 is essential for early development, and further insight into EVI1 function will help to define its role in maintenance of the stem cell phenotype. In view of the clinical relevance of aberrantly high expression of EVI1 for malignant proliferation, better understanding of EVI1 functions will potentially also open novel therapeutic perspectives for EVI1 overexpressing malignancies. Our mass spectrometry- and antibody-based analysis of *endogenously* expressed EVI1 in SB1690CB AML cells confirms phosphorylation of the carboxy-terminal SQS site (S858 and S860). Carboxy-terminal phosphorylation of EVI1 has previously been listed in global phosphoproteomic and targeted studies (29,40,48), but has not been further characterized or functionally assessed in any detail. We detected EVI1 not phosphorylated at this motif, phosphorylated at S860, or doubly phosphorylated at S860 and S858 after induction of DNA damage. Building on previously published studies as above (29,40,48), our data imply that the S860 phosphorylation is likely to be present during normal cell proliferation, while the double phosphorylation on S860 and S858 is promoted via ATM, in particular in response to genotoxic stress. Reduction of the level of double phosphorylation with an ATM-inhibitor is in line with the recognition of the SQS-sequence as a specific ATM motif (40). Given the role of ATM in stem cell self-renewal (43), and EVI1 for maintenance of the stem cell phenotype (1), we hypothesized a role of the carboxy-terminal SQS motif for this EVI1 function, which we investigated by EVI1 site directed mutagenesis and gene transfer. We first show that the modulatory effect mediated by EVI1 on transcriptional patterns the presence genotoxic stress is dependent on the carboxy-terminal SQS motif. Aware of the limitations using the HEK293 model for investigating EVI1-target genes, we noted that known EVI1-affected transcripts such as *ALDOC* (18) were amongst significantly differentially expressed transcripts also in our model, and further investigations in respect of EVI1-modulated gene expression in particular in the presence of genotoxic stress, which in our model affected distinct cellular pathways, will be important to understand EVI1-mediated transcriptional modulation. In addition, in a clinical context modulation of EVI1-mediated gene expression by genotoxic stress and ATM, for example in response to chemotherapy, is likely to be relevant for gene expression patterns in EVI1-expressing leukaemia and solid tumors also in patient samples. We demonstrated the relevance of the carboxy-terminal SQS phosphorylation with respect to EVI1-mediated transformation using Rat-1 fibroblasts, for which in the presence of genotoxic stress only phosphorylatable EVI1-WT sustains transformation, while EVI1-AQA lacking the ATM consensus site had reduced transforming ability. In murine haematopoietic progenitor cell replating, modelling the EVI1-function in early hematopoiesis, mutation of the carboxy-terminal ATM motif SQS to AQA significantly reduced re-plating capacity. ATM is required for the regulation of oxidative stress for self-renewal of hematopoietic cells (43), and *EVI1* expression is high in early haematopoietic cells (49). Thus, EVI1 mediated hematopoietic self-renewal or maintenance capacity is likely linked by an ATM-EVI1 axis, which is supported by the demonstration of reduced self-renewal mediated by EVI1-AQA, which lacks the ATM consensus site. But how is this achieved when mutation of the carboxy terminal SQS motif does affect DNA-binding and zinc finger motifs of EVI1, and does not alter gene promotor repression? We show that the EVI1-interaction with CtBP1 is dynamically and finely modulated by the ATM-mediated EVI1-SQS phosphorylation and enhanced by genotoxic stress. The interaction with CtBP1 is essential for EVI1 function with respect to transformation and leukaemogenesis in murine haematopoietic progenitor cells (27,50), and modulated by ATM without directly affecting previously described CtBP1 binding regions, which involve amino acids 553–559 and 584–590 (27). This might involve charge-mediated affinity between proteins, altered tertiary structure, or posttranslational modification of CtBP1 or other proteins, which we are investigating. Data presented here support a concept by which EVI1 phosphorylation links ATM and the DNA damage response to essential EVI1 protein interactions for transcriptional regulation, transformation and self-renewal. This novel aspect of EVI1 function, in particular with respect to transformation and self-renewal, is therapeutically relevant for EVI1-overexpressing malignancies, as chemotherapy induced genotoxicity might detrimentally sustain EVI1 function. Inhibition of ATM with an orally applicable compound (51) is currently subject to phase 1 clinical evaluation and has shown activity in a murine leukaemia model (52). ATM inhibition or therapeutic interference of the enhanced EVI1 interaction with CtBP1 after genotoxic stress might be of particular benefit in EVI1 overexpressing malignancies, and this will be subject to further work.

## DATA AVAILABILITY

Gene expression data from RNA*seq* experiments are available on GEO (submission No GSE115643).

## Supplementary Material

Supplementary Data

## References

[1] Goyama S., Yamamoto G., Shimabe M., Sato T., Ichikawa M., Ogawa S., Chiba S., Kurokawa M. Evi-1 is a critical regulator for hematopoietic stem cells and transformed leukemic cells. Cell Stem Cell. 2008; 3:207–220. 18682242 10.1016/j.stem.2008.06.002

[2] Konantz M., Alghisi E., Muller J.S., Lenard A., Esain V., Carroll K.J., Kanz L., North T.E., Lengerke C. Evi1 regulates Notch activation to induce zebrafish hematopoietic stem cell emergence. EMBO J. 2016; 35:2315–2331. 27638855 10.15252/embj.201593454 PMC5090218

[3] Kataoka K., Sato T., Yoshimi A., Goyama S., Tsuruta T., Kobayashi H., Shimabe M., Arai S., Nakagawa M., Imai Y. Evi1 is essential for hematopoietic stem cell self-renewal, and its expression marks hematopoietic cells with long-term multilineage repopulating activity. J. Exp. Med. 2011; 208:2403–2416. 22084405 10.1084/jem.20110447 PMC3256960

[4] Buonamici S., Li D., Chi Y., Zhao R., Wang X., Brace L., Ni H., Saunthararajah Y., Nucifora G. EVI1 induces myelodysplastic syndrome in mice. J. Clin. Invest. 2004; 114:713–719. 15343390 10.1172/JCI21716 PMC514587

[5] Bartholomew C., Kilbey A., Clark A.M., Walker M. The Evi-1 proto-oncogene encodes a transcriptional repressor activity associated with transformation. Oncogene. 1997; 14:569–577. 9053855 10.1038/sj.onc.1200864

[6] Groschel S., Lugthart S., Schlenk R.F., Valk P.J., Eiwen K., Goudswaard C., van Putten W.J., Kayser S., Verdonck L.F., Lubbert M. High EVI1 expression predicts outcome in younger adult patients with acute myeloid leukemia and is associated with distinct cytogenetic abnormalities. J. Clin. Oncol. 2010; 28:2101–2107. 20308656 10.1200/JCO.2009.26.0646

[7] Lugthart S., van Drunen E., van Norden Y., van Hoven A., Erpelinck C.A., Valk P.J., Beverloo H.B., Lowenberg B., Delwel R. High EVI1 levels predict adverse outcome in acute myeloid leukemia: prevalence of EVI1 overexpression and chromosome 3q26 abnormalities underestimated. Blood. 2008; 111:4329–4337. 18272813 10.1182/blood-2007-10-119230

[8] Stein S., Ott M.G., Schultze-Strasser S., Jauch A., Burwinkel B., Kinner A., Schmidt M., Kramer A., Schwable J., Glimm H. Genomic instability and myelodysplasia with monosomy 7 consequent to EVI1 activation after gene therapy for chronic granulomatous disease. Nat. Med. 2010; 16:198–204. 20098431 10.1038/nm.2088

[9] Meyer S., Bristow C., Wappett M., Pepper S., Whetton A.D., Hanenberg H., Neitzel H., Wlodarski M.W., Ebell W., Tönnies H. Fanconi anemia (FA)-associated 3q gains in leukemic transformation consistently target EVI1, but do not affect low TERC expression in FA. Blood. 2011; 117:6047–6050. 21636719 10.1182/blood-2011-03-343897

[10] Meyer S., Fergusson W.D., Whetton A.D., Moreira-Leite F., Pepper S.D., Miller C., Saunders E.K., White D.J., Will A.M., Eden T. Amplification and translocation of 3q26 with overexpression of EVI1 in Fanconi anemia-derived childhood acute myeloid leukemia with biallelic FANCD1/BRCA2 disruption. Genes Chromosomes Cancer. 2007; 46:359–372. 17243162 10.1002/gcc.20417

[11] Dutta P., Bui T., Bauckman K.A., Keyomarsi K., Mills G.B., Nanjundan M. EVI1 splice variants modulate functional responses in ovarian cancer cells. Mol. Oncol. 2013; 7:647–668. 23517670 10.1016/j.molonc.2013.02.008 PMC3805042

[12] Koos B., Bender S., Witt H., Mertsch S., Felsberg J., Beschorner R., Korshunov A., Riesmeier B., Pfister S., Paulus W. The transcription factor evi-1 is overexpressed, promotes proliferation, and is prognostically unfavorable in infratentorial ependymomas. Clin. Cancer Res. 2011; 17:3631–3637. 21493867 10.1158/1078-0432.CCR-11-0175

[13] Wang H., Schaefer T., Konantz M., Braun M., Varga Z., Paczulla A.M., Reich S., Jacob F., Perner S., Moch H. Prominent oncogenic roles of EVI1 in breast carcinoma. Cancer Res. 2017; 77:2148–2160. 28209621 10.1158/0008-5472.CAN-16-0593

[14] Queisser A., Hagedorn S., Wang H., Schaefer T., Konantz M., Alavi S., Deng M., Vogel W., von Massenhausen A., Kristiansen G. Ecotropic viral integration site 1, a novel oncogene in prostate cancer. Oncogene. 2017; 36:1573–1584. 27617580 10.1038/onc.2016.325

[15] Krivtsov A.V., Figueroa M.E., Sinha A.U., Stubbs M.C., Feng Z., Valk P.J., Delwel R., Dohner K., Bullinger L., Kung A.L. Cell of origin determines clinically relevant subtypes of MLL-rearranged AML. Leukemia. 2013; 27:852–860. 23235717 10.1038/leu.2012.363 PMC4693300

[16] Regha K., Assi S.A., Tsoulaki O., Gilmour J., Lacaud G., Bonifer C. Developmental-stage-dependent transcriptional response to leukaemic oncogene expression. Nat. Commun. 2015; 6:7203. 26018585 10.1038/ncomms8203 PMC4458875

[17] Stevens A., Hanson D., de Leonibus C., Whatmore A., Donn R., White D.J., Liu J., van den Heuvel-Eibrink M.M., Saha V., Clayton P.E. EVI1 expression in childhood acute lymphoblastic leukaemia is not restricted to MLL and BCR/ABL rearrangements and is influenced by age. Blood Cancer J. 2014; 4:e179. 24464103 10.1038/bcj.2013.76 PMC3913945

[18] Fenouille N., Bassil C.F., Ben-Sahra I., Benajiba L., Alexe G., Ramos A., Pikman Y., Conway A.S., Burgess M.R., Li Q. The creatine kinase pathway is a metabolic vulnerability in EVI1-positive acute myeloid leukemia. Nat. Med. 2017; 23:301–313. 28191887 10.1038/nm.4283 PMC5540325

[19] Kustikova O.S., Schwarzer A., Stahlhut M., Brugman M.H., Neumann T., Yang M., Li Z., Schambach A., Heinz N., Gerdes S. Activation of Evi1 inhibits cell cycle progression and differentiation of hematopoietic progenitor cells. Leukemia. 2013; 27:1127–1138. 23212151 10.1038/leu.2012.355

[20] Delwel R., Funabiki T., Kreider B.L., Morishita K., Ihle J.N. Four of the seven zinc fingers of the Evi-1 myeloid-transforming gene are required for sequence-specific binding to GA(C/T)AAGA(T/C)AAGATAA. Mol. Cell. Biol. 1993; 13:4291–4300. 8321231 10.1128/mcb.13.7.4291-4300.1993 PMC359982

[21] Funabiki T., Kreider B.L., Ihle J.N. The carboxyl domain of zinc fingers of the Evi-1 myeloid transforming gene binds a consensus sequence of GAAGATGAG. Oncogene. 1994; 9:1575–1581. 8183551

[22] Izutsu K., Kurokawa M., Imai Y., Maki K., Mitani K., Hirai H. The corepressor CtBP interacts with Evi-1 to repress transforming growth factor beta signaling. Blood. 2001; 97:2815–2822. 11313276 10.1182/blood.v97.9.2815

[23] Goyama S., Nitta E., Yoshino T., Kako S., Watanabe-Okochi N., Shimabe M., Imai Y., Takahashi K., Kurokawa M. EVI-1 interacts with histone methyltransferases SUV39H1 and G9a for transcriptional repression and bone marrow immortalization. Leukemia. 2010; 24:81–88. 19776757 10.1038/leu.2009.202

[24] Chi Y., Senyuk V., Chakraborty S., Nucifora G. EVI1 promotes cell proliferation by interacting with BRG1 and blocking the repression of BRG1 on E2F1 activity. J. Biol. Chem. 2003; 278:49806–49811. 14555651 10.1074/jbc.M309645200

[25] Yoshimi A., Goyama S., Watanabe-Okochi N., Yoshiki Y., Nannya Y., Nitta E., Arai S., Sato T., Shimabe M., Nakagawa M. Evi1 represses PTEN expression and activates PI3K/AKT/mTOR via interactions with polycomb proteins. Blood. 2011; 117:3617–3628. 21289308 10.1182/blood-2009-12-261602

[26] Nitta E., Izutsu K., Yamaguchi Y., Imai Y., Ogawa S., Chiba S., Kurokawa M., Hirai H. Oligomerization of Evi-1 regulated by the PR domain contributes to recruitment of corepressor CtBP. Oncogene. 2005; 24:6165–6173. 15897867 10.1038/sj.onc.1208754

[27] Palmer S., Brouillet J.P., Kilbey A., Fulton R., Walker M., Crossley M., Bartholomew C. Evi-1 transforming and repressor activities are mediated by CtBP co-repressor proteins. J. Biol. Chem. 2001; 276:25834–25840. 11328817 10.1074/jbc.M102343200

[28] White D.J., Unwin R.D., Bindels E., Pierce A., Teng H.Y., Muter J., Greystoke B., Somerville T.D., Griffiths J., Lovell S. Phosphorylation of the leukemic oncoprotein EVI1 on serine 196 modulates DNA binding, transcriptional repression and transforming ability. PLoS One. 2013; 8:e66510. 23776681 10.1371/journal.pone.0066510 PMC3680417

[29] Bard-Chapeau E.A., Gunaratne J., Kumar P., Chua B.Q., Muller J., Bard F.A., Blackstock W., Copeland N.G., Jenkins N.A. EVI1 oncoprotein interacts with a large and complex network of proteins and integrates signals through protein phosphorylation. Proc. Natl. Acad. Sci. U.S.A. 2013; 110:E2885–E2894. 23858473 10.1073/pnas.1309310110 PMC3732971

[30] Gulden M., Jess A., Kammann J., Maser E., Seibert H. Cytotoxic potency of H2O2 in cell cultures: impact of cell concentration and exposure time. Free Rad. Biol. Med. 2010; 49:1298–1305. 20673847 10.1016/j.freeradbiomed.2010.07.015

[31] Unwin R.D., Griffiths J.R., Leverentz M.K., Grallert A., Hagan I.M., Whetton A.D. Multiple reaction monitoring to identify sites of protein phosphorylation with high sensitivity. Mol. Cell. Proteomics: MCP. 2005; 4:1134–1144. 15923565 10.1074/mcp.M500113-MCP200

[32] Liu Y., Chen L., Ko T.C., Fields A.P., Thompson E.A. Evi1 is a survival factor which conveys resistance to both TGFbeta- and taxol-mediated cell death via PI3K/AKT. Oncogene. 2006; 25:3565–3575. 16462766 10.1038/sj.onc.1209403

[33] Konantz M., Andre M.C., Ebinger M., Grauer M., Wang H., Grzywna S., Rothfuss O.C., Lehle S., Kustikova O.S., Salih H.R. EVI-1 modulates leukemogenic potential and apoptosis sensitivity in human acute lymphoblastic leukemia. Leukemia. 2013; 27:56–65. 22828445 10.1038/leu.2012.211

[34] Schambach A., Bohne J., Chandra S., Will E., Margison G.P., Williams D.A., Baum C. Equal potency of gammaretroviral and lentiviral SIN vectors for expression of O6-methylguanine-DNA methyltransferase in hematopoietic cells. Mol. Ther. 2006; 13:391–400. 16226060 10.1016/j.ymthe.2005.08.012

[35] Ding X., Ray Chaudhuri A., Callen E., Pang Y., Biswas K., Klarmann K.D., Martin B.K., Burkett S., Cleveland L., Stauffer S. Synthetic viability by BRCA2 and PARP1/ARTD1 deficiencies. Nat. Commun. 2016; 7:12425. 27498558 10.1038/ncomms12425 PMC4979061

[36] Aken B.L., Achuthan P., Akanni W., Amode M.R., Bernsdorff F., Bhai J., Billis K., Carvalho-Silva D., Cummins C., Clapham P. Ensembl 2017. Nucleic Acids Res. 2017; 45:D635–D642. 27899575 10.1093/nar/gkw1104 PMC5210575

[37] Somervaille T.C., Matheny C.J., Spencer G.J., Iwasaki M., Rinn J.L., Witten D.M., Chang H.Y., Shurtleff S.A., Downing J.R., Cleary M.L. Hierarchical maintenance of MLL myeloid leukemia stem cells employs a transcriptional program shared with embryonic rather than adult stem cells. Cell Stem Cell. 2009; 4:129–140. 19200802 10.1016/j.stem.2008.11.015 PMC2670853

[38] Cante-Barrett K., Mendes R.D., Smits W.K., van Helsdingen-van Wijk Y.M., Pieters R., Meijerink J.P. Lentiviral gene transfer into human and murine hematopoietic stem cells: size matters. BMC Res. Notes. 2016; 9:312. 27306375 10.1186/s13104-016-2118-z PMC4910193

[39] Dunn K.W., Kamocka M.M., McDonald J.H. A practical guide to evaluating colocalization in biological microscopy. Am. J. Physiol. Cell Physiol. 2011; 300:C723–C742. 21209361 10.1152/ajpcell.00462.2010 PMC3074624

[40] Matsuoka S., Ballif B.A., Smogorzewska A., McDonald E.R. 3rd, Hurov K.E., Luo J., Bakalarski C.E., Zhao Z., Solimini N., Lerenthal Y. ATM and ATR substrate analysis reveals extensive protein networks responsive to DNA damage. Science. 2007; 316:1160–1166. 17525332 10.1126/science.1140321

[41] Bard-Chapeau E.A., Jeyakani J., Kok C.H., Muller J., Chua B.Q., Gunaratne J., Batagov A., Jenjaroenpun P., Kuznetsov V.A., Wei C.L. Ecotopic viral integration site 1 (EVI1) regulates multiple cellular processes important for cancer and is a synergistic partner for FOS protein in invasive tumors. Proc. Natl. Acad. Sci. U.S.A. 2012; 109:2168–2173. 22308434 10.1073/pnas.1119229109 PMC3277513

[42] Takahashi S., Licht J.D. The human promyelocytic leukemia zinc finger gene is regulated by the Evi-1 oncoprotein and a novel guanine-rich site binding protein. Leukemia. 2002; 16:1755–1762. 12200691 10.1038/sj.leu.2402682

[43] Ito K., Hirao A., Arai F., Matsuoka S., Takubo K., Hamaguchi I., Nomiyama K., Hosokawa K., Sakurada K., Nakagata N. Regulation of oxidative stress by ATM is required for self-renewal of haematopoietic stem cells. Nature. 2004; 431:997–1002. 15496926 10.1038/nature02989

[44] Chinnadurai G. The transcriptional corepressor CtBP: a foe of multiple tumor suppressors. Cancer Res. 2009; 69:731–734. 19155295 10.1158/0008-5472.CAN-08-3349 PMC4367538

[45] Di L.J., Byun J.S., Wong M.M., Wakano C., Taylor T., Bilke S., Baek S., Hunter K., Yang H., Lee M. Genome-wide profiles of CtBP link metabolism with genome stability and epithelial reprogramming in breast cancer. Nat. Commun. 2013; 4:1449. 23385593 10.1038/ncomms2438 PMC3768144

[46] Jeyasekharan A.D., Ayoub N., Mahen R., Ries J., Esposito A., Rajendra E., Hattori H., Kulkarni R.P., Venkitaraman A.R. DNA damage regulates the mobility of Brca2 within the nucleoplasm of living cells. Proc. Natl. Acad. Sci. U.S.A. 2010; 107:21937–21942. 21098284 10.1073/pnas.1009577107 PMC3003017

[47] Reuter M., Zelensky A., Smal I., Meijering E., van Cappellen W.A., de Gruiter H.M., van Belle G.J., van Royen M.E., Houtsmuller A.B., Essers J. BRCA2 diffuses as oligomeric clusters with RAD51 and changes mobility after DNA damage in live cells. J. Cell Biol. 2015; 208:857. 25778924 10.1083/jcb.20140501402182015c PMC4362460

[48] Olsen J.V., Blagoev B., Gnad F., Macek B., Kumar C., Mortensen P., Mann M. Global, in vivo, and site-specific phosphorylation dynamics in signaling networks. Cell. 2006; 127:635–648. 17081983 10.1016/j.cell.2006.09.026

[49] Cabezas-Wallscheid N., Klimmeck D., Hansson J., Lipka D.B., Reyes A., Wang Q., Weichenhan D., Lier A., von Paleske L., Renders S. Identification of regulatory networks in HSCs and their immediate progeny via integrated proteome, transcriptome, and DNA methylome analysis. Cell Stem Cell. 2014; 15:507–522. 25158935 10.1016/j.stem.2014.07.005

[50] Senyuk V., Chakraborty S., Mikhail F.M., Zhao R., Chi Y., Nucifora G. The leukemia-associated transcription repressor AML1/MDS1/EVI1 requires CtBP to induce abnormal growth and differentiation of murine hematopoietic cells. Oncogene. 2002; 21:3232–3240. 12082639 10.1038/sj.onc.1205436

[51] Pike K.G., Barlaam B., Cadogan E., Campbell A., Chen Y., Colclough N., Davies N.L., DeAlmeida C., Degorce S.L., Didelot M. The identification of potent, selective and orally available inhibitors of ataxia telangiectasia mutated (ATM) Kinase: The discovery of AZD0156 (8-{6-[3-(dimethylamino)propoxy]pyridin-3-yl}-3-methyl-1-(tetrahydro-2H-pyran-4-y l)-1,3-dihydro-2H-imidazo[4,5-c]quinolin-2-one). J. Med. Chem. 2018; 61:3823–3841. 29683659 10.1021/acs.jmedchem.7b01896

[52] Morgado-Palacin I., Day A., Murga M., Lafarga V., Anton M.E., Tubbs A., Chen H.T., Ergan A., Anderson R., Bhandoola A. Targeting the kinase activities of ATR and ATM exhibits antitumoral activity in mouse models of MLL-rearranged AML. Sci. Signal. 2016; 9:ra91. 27625305 10.1126/scisignal.aad8243 PMC5066844

