# Supplementary material for: EVI1 carboxy-terminal phosphorylation is ATM-mediated and sustains transcriptional modulation and self-renewal via enhanced CtBP1 association
Source: Nucleic Acids Res. 2018 Jun 25;46(15):7662–74. doi: 10.1093/nar/gky536 (PMC6125627; doi:10.1093/nar/gky536)

EVI1 carboxy-terminal phosphorylation is ATM-mediated and sustains transcriptional modulation and self-renewal via enhanced CtBP1 association

Roberto Paredes *et al*

Supplementary Materials and Methods, Table 1, 2, and 3, legend to supplementary Table 4 (Excel file) and supplementary Figures with legends

Supplementary Materials and Methods

Western blot quantification

Chemo luminescent western blot signals were acquired in a Gel Doc XRS System using the Quantity One software (BioRad). Digital files were generated form recordings of sequential exposures in one minute intervals for 30 minutes (Quantity One .1sc format). Image analysis was carried out using the ImageJ software using the LOCI Bio-format importer plug-in (https://www.openmicroscopy.org/site) to open .1sc files. Images were processed in HiLo LUT to avoid saturated signals. To quantify the signal of specific bands, integrated density was measured over regions of interest (ROI) over each imprinted band using the Analyze tool of ImageJ (**Supplementary Figure 2**). Values of EVI1-IPs were normalized against the EVI1 inputs, and values of the CtBP1 co-IP against the EVI1-IP.

Protein sequences alignment

EVI1 protein sequences from the NCBI database were aligned using ClustalW2 or Clustal Omega programs (1), and analyzed and illustrated using the Uniprot knowledgebase (2) and the Jalview software (3).

Reporter gene assays

Reporter gene assays were carried out in HEK293T cells as described before (4). Cells were plated in 6-well plates and co-transfected with pCMV-EVI1-flag effector plasmids (EVI1-WT or EVI1-AQA), PLZF-luciferase or FOS-luciferase and TK-renilla luciferase reporter plasmid. Comparable expression of EVI1-WT and EVI1-AQA in HEK293 cells was monitored by RT-PCR (see below) **(Supplementary Figure 4)**. After 48 hours cells were lysed and firefly and renilla luciferase activities were assayed using the Dual-luciferase reporter assay (Promega) measured on a Victor plate reader (Perkin Elmer).

Real time PCR

Real time PCR (RT-PCR) was carried out with cDNA synthesised from RNA using the Transcriptor First Strand cDNA Synthesis Kit (Roche) as per manufacturer's instruction. cDNA was quantified using Qubit™ dsDNA HS Assay Kit (Invitrogen) with Qubit™ 4 Fluorometer (Invitrogen). Of a panel of 10 housekeeper transcripts *YWAZ* and *β-Actin* were selected for minimal variations between conditions and averaged. Transcripts were detected on a QuantStudio 5 Real-Time PCR System (Applied Biosystems) using TaqMan™ Fast Advanced Master Mix (Applied Biosystems) using primer oligonucleoties and Probes, mostly from the Roche Universal Library as listed in **Supplementary Table 1**. EVI1 primers and probes were used as in Gröschel et al. (5). The ΔΔCt method was used to calculate fold change values in comparison to untransduced controls using the mean of housekeeper CTs (6).

Immunofluorescence (IF) analysis

Suspension leukaemia cells SB1690CB cells were spun at 200 rpm for 2 min (Cytospin 2, Shandon) onto POLYSINE slides (VWR International). HEK293T cells were seeded and transfected in glass-bottomed dishes coated with poly-D-lysine (MatTek). Cells were fixed with methanol-free 4% formaldehyde (Thermo Scientific), washed in PBS and blocked with 5% goat normal serum (Cell Signaling Technology) and 0.3% Triton-X100 (Sigma) containing PBS. □H2AX, CtBP1 and EVI1 antibodies were used with secondary antibodies following standard procedures in PBS containing 0.1% BSA (Cell Signaling Technology) and 0.3% Triton-X100. Conventional fluorescent microscopy was performed in an Olympus BX51 microscope using an UPlanSApo 100X oil immersion lens and the Q-capturePro7 imaging system (Retiga 6000 camera, QImaging). Single confocal plane and sequential channel acquisitions were performed in a Fluoview1000 confocal system (Olympus), using a 60X UPLSAPO oil immersion lens. To determine levels of co-localisation of EVI1 and CtBP1 signals, Pearson product-moment correlation coefficient (Pearson's coefficient (7)) was used to measure the linear signal correlation (dependence) between the EVI1 and CtBP1 IF signals. Person's coefficient ranged from 1 (total positive correlation) to −1 (total negative correlation). Regions of interest (ROI) covering the entire positively stained area were analysed in 100 cells per condition with the co-localisation plug-in of the ImageJ software. To discard signal saturated ROIs, the images were analysed using HiLo (High-Low) intensity Look Up Table (LUT). Pearson coefficients were plotted either in a dispersion graph (cell distribution in a single experiment) or as average from at least three biological replicates. One-way Analysis Of the Variance (ANOVA) with the

Tukey post-test statistical analysis was used to compare the means (GraphPad Prism).

<u>Supplementary Table 1</u>:

Antibodies (in addition to the antibodies detailed in the main paper):

**Antigen**	**Company**	**Catalogue No**
p53	Abcam	#Ab1101
Phospho-p53 (Ser15)	Cell Signaling Technology	#9284
Histone H_3	Cell Signaling Technology	#9715
Phospho-Histone H2A.X (Ser139)	Cell Signaling Technology	#9718
Phospho-Histone H2A.X (Ser139)	NOVUS	#NB200-783, 56
CtBP1	BD Biosciences	#612042
EVI1	(C50E12) Cell Signaling Technology	#2593
FLAG	Anti-FLAG® M2 Magnetic Beads Sigma	# M882
Mouse IgG	Anti-mouse IgG HRP Linked Whole Ab, Amersham ECL, GE Healthcare	#NA931V
Rabbit IgG	Anti-rabbit IgG HRP Linked Whole Ab, Amersham ECL, GE Healthcare	#NA9340V
Mouse IgG	Goat anti-mouse IgG (H+L) Secondary antibody, Alexa Fluor 594 conjugated, Life Technologies	#A-11032
Rabbit IgG	Goat anti-rabbit IgG (H+L) Secondary antibody, Alexa Fluor 488 conjugated, Life Technologies	#A-11034

Supplementary Table 2: Primer and probe oligonucleotide sequences for RT-PCR

Transcript	Primer 5'-3'	Primer 3'-5'	Probe (*Roche Universal probe library)
ALDOC	GGATCAGAACCCGAGCTGT	TACGAGTGAGGCATGGTGAC	5*
ABCD2	TGTCCATCTCTATCACATAGTTCAAA	ACAGGACATCTTTCCAGTCCA	8*
ADGRB2	AGCCACTGGAACCCCATC	TTTTTCAGCAGCAAAATCCAC	23*
HYDIN	TGGCGCTCTTAATTACAGCAA	GCCCAAAGTCCACCTCTGTA	39*
MOV10	ACCTGGGACCCTGTGGAC	CGGCTCTCAGTCACTCCAG	64*
SETX	TTTTTCCCTTTGATGGTATTGAA	CTATTTGGAGAGTTGAGCCATTC	59*
TMEM222	CCTGCTCTACGGGAAGTACG	TGATGCCCAGGAGAAGGAT	71*
TOM1L1	CCAGAAGGAAGCCACCAATA	TGGGACTTAGGTCGAGGAGA	29*
βACTIN	CCAACCGCGAGAAGATGA	CCAGAGGCGTACAGGGATAG	64*
YWHAZ	GATCCCCAATGCTTCACAAG	TGCTTGTTGTGACTGATCGAC	30*
EVI1	AGTGCCCTGGAGATGAGTTG	TTTGAGGCTATCTGTGAAGTGC	Dual labelled probe: 5' FAM CCCCAGTGAGGTATA AAGAGGA 3' TAM

Supplementary Table 3:

Site directed mutagenesis primers

**Human EVI1 SQS to AQA**	
S858A_S860A_FW	5'GAACAGCAACCATGGCGCCCAAGTCCCCAGGAATGTGG3'
S858A_S860A_RV	5'CCACATTCCTGGGAGCTTGGGCGCCATGGTTGCTGTTC3'
**Mouse Codon optimized EVI1 SQS to AQA**	
AQA_FW	5'TCCATGTTCCGGGGGGCCTGGGCGCCGTGGTTGCTGTT3'
AQA_RV	5'AACAGCAACCACGGCGCCCAGGCCCCCCGGAACATGGA3'

Supplementary Table 4 **(attached excel file):**

Supplementary Table 4, Sheet 1. *Effect of EVI1-WT and EVI1-AQA on transcription 1*.

Transcripts identified by the group ANOVA (n=1306) are shown in heatmap order (Figure 3B) and listed in column 1.

Individual two o group comparisons are as described in the column headers. The two group comparison p-values are presented as a blue heatmap (deep blue as highly significant and grey as non-significant). The expression ration between the two groups is shown as a heatmap with intensity of red indicating increased fold change and intensity of green indicating reduced fold change.

Gene ID column is coloured to highlight the groups defined in Figure 3B.

Green Gene ID: Cluster 1, 139 transcripts. Red -shaded transcript name: arrow in figure 3B

Red Gene ID: Cluster 2, 328 transcripts. Green shaded transcript name: subcluster in 2B in figure 3B

UN= untransfected, EV = Empty vector.

Supplementary Table 4, Sheet 2. *Effect of EVI1-WT and EVI1-AQA on transcription* 2

Detailed two group comparison of transcripts significantly upregulated or repressed ($p<0.05$) in transcripts identified by group ANOVA (Supplemental Table S1) comparing EVI1 WT vs Emtpy vector (EV) and EVI1-AQA vs EV untreated and in the presence of H_2O_2.

In columns A-T: green = downregulated, red = upregulated.

In columns A & F: pink = overlap

In columns K & P: yellow = overlap

Overlaps visualised as Venn Diagrams, red = upregulated genes, green = downregulated genes

Supplementary Table 4, sheet 3: ***Effect of EVI1-WT and EVI1-AQA on transcription 3.*** Pathway analysis of differentially expressed genes applying a right sided Fisher's exact test (Ingenuity Pathway Analysis, Qiagen). Using the group ANOVA data (Supplemental Table S1), the transcripts associated with the two group comparisons presented in Supplemental S2 are analysed to determine significantly associated biological pathways. In columns B-E the -log(p-value) of association is presented (1.3 is equivalent to p=0.05), these data are shown in the figure and an orange dotted line is used to mark -log(p-value) = 1.3.

Supplementary Table 4, sheet 4: ***Effect of EVI1-WT and EVI1-AQA on transcription 4:*** **Real- time PCR confirmation of selected genes.** Real time PCR of selected genes as indicated. Shown are ΔΔCT values compared to untransfected cells untreated (bright grey) and H_2O_2 treated (dark grey). Red dots indicate fold change from RNA seq data (sheet 1).

Legends to Supplementary Figures

Supplementary Figure 1: A: Validation of the anti-pS860-EVI1 antibody for western blot. EVI1 was immunoprecipated from EVI1 expressing SB1690CB (SB) untreated or non-EVI1 expressing OCI-AML5 (OCI) cells and resolved by electrophoresis. The blotted membrane was incubated with the primary antibody in the presence or

absence of blocking peptides as indicated. Secondary antibody and signal developing was carried out using standard methodologies. **B:** Validation of the anti-p558/pS860-EVI1 antibody for western blot. EVI1 was immunoprecipated from SB1690CB cells after irradiation (5Gy). The blotted membrane was incubated with the primary anti-p558/pS860-EVI1 antibody in the presence and in the absence of the indicated blocking peptides. Secondary antibody and signal developing was carried out using standard methodologies.

Supplementary Figure 2: Western blot chemo luminescence quantification using the Gel Doc XRS System by ImageJ: **A:** Raw .sc1 files related to figure 6C, opened at ImageJ as greyscale (left panels) and HiLo LUT (right panels). Red mask denotes signal-saturated pixels. A dashed line indicates the separations between the two blots. Flag (EVI1) associated signal (yellow oblongs) and CtBP1-associated signals (green oblongs) were quantified during indicated exposure time and plotted to calibrate signal saturation point (**B** and **C**). Plots B and D of the figure 7 were built using exposures time lower than 5 minutes and non-saturated signal.

Supplementary Figure 3: Sanger sequencing, confirming mutagenesis of the c-terminal SQS motif to AQA of human and codon optimized mouse EVI1 vector.

Supplementary Figure 4: Monitoring EVI1 expression in transfected HEK293 cells. Total protein lysates of EVI1 transfected HEK29T cells corresponding to figures 3A and 6A were analyzed by RT-PCR **(A)** or western blot **(B)** for EVI1 expression. Ponceau-S stain for protein loading control (B, lower panel). T-test, n.s.: not significant.

Supplementary Figure 5: Rat-1 fibroblast colony forming assay. A: Constructs for Rat-1 fibroblast transduction. **B:** Western blot showing comparable EVI1-WT and EVI1-AQA mutant expression in stably transduced Rat-1 fibroblast. **C:** Maintenance of GFP expression during colony-forming assay assessed by epifluorescent acquisition merged with bright field images. **D.** Morphology of typical Rat-1 colony (red arrowheads) after 14 days in culture. Colony size after transfection in the presence of damage non-specific and not significantly different in MSCV- compared with EVI1-transfected cells (data not shown). **E:** H_2O_2 or irradiation induced γH2AX foci formation confirming activation of the DNA damage response in Rat-1 fibroblast (white arrowheads). DAPI for nuclear stain.

Supplementary Figure 6: Lentivirus production, mouse $cKit^+$ cells transduction and sorting. A: Transduced $cKit^+$ cells were analyzed by flow cytometry, gated and sorted by GFP emission (green portion of the cell count histograms), showing percentages of transduced cell **(B)** comparing EVI1 WT and the EVI1 AQA mutant. (n.s.=not significant, T-test). **C.** Dose response for mouse $cKit^+$ cells for H_2O_2, for IC_{50} calculation used in the colony formation assay. **D.** H_2O_2 activation of the DNA damage response in mouse $cKit^+$ cells visualised by □H2AX foci. **E.** Typical $cKit^+$ colony morphology in methylcellulose-based medium after 7 days in culture. **F.** $cKit^+$ single colony May-Grünwald Giemsa stain (right hand side panels) showing presence of active cell division (mitotic cells, arrows) and differentiated cells (arrows) in the same colonies. Direct bright field microscopy (left hand side panels) was used to score colony morphology.

Supplementary Figure 7: Validation of EVI1 antibody and genotoxic stress markers for immunofluorescence (IF) staining. A: EVI1 and CtBP1 immunofluorescence in EVI1 expressing SB1690CB AML cells, with OCI-AML5 cells as EVI1 negative controls. **B**: γH2AX foci formation after cell irradiation, and **C**: γH2AX foci formation after H_2O_2 treatment in transfected HEK293 cells (see figure 3 and figure 6, main manuscript).

Supplementary references:

1. Sievers, F., Wilm, A., Dineen, D., Gibson, T.J., Karplus, K., Li, W., Lopez, R., McWilliam, H., Remmert, M., Soding, J. *et al.* (2011) Fast, scalable generation of high-quality protein multiple sequence alignments using Clustal Omega. *Molecular systems biology*, **7**, 539.
2. The UniProt, C. (2017) UniProt: the universal protein knowledgebase. *Nucleic acids research*, **45**, D158-D169.
3. Waterhouse, A.M., Procter, J.B., Martin, D.M., Clamp, M. and Barton, G.J. (2009) Jalview Version 2--a multiple sequence alignment editor and analysis workbench. *Bioinformatics*, **25**, 1189-1191.
4. White, D.J., Unwin, R.D., Bindels, E., Pierce, A., Teng, H.Y., Muter, J., Greystoke, B., Somerville, T.D., Griffiths, J., Lovell, S. *et al.* (2013) Phosphorylation of the leukemic oncoprotein EVI1 on serine 196 modulates DNA binding, transcriptional repression and transforming ability. *PloS one*, **8**, e66510.
5. Groschel, S., Lugthart, S., Schlenk, R.F., Valk, P.J., Eiwen, K., Goudswaard, C., van Putten, W.J., Kayser, S., Verdonck, L.F., Lubbert, M. *et al.* (2010) High EVI1 expression predicts outcome in younger adult patients with acute myeloid leukemia and is associated with distinct cytogenetic abnormalities. *Journal of clinical oncology : official journal of the American Society of Clinical Oncology*, **28**, 2101-2107.
6. Livak, K.J. and Schmittgen, T.D. (2001) Analysis of relative gene expression data using real-time quantitative PCR and the 2(-Delta Delta C(T)) Method. *Methods*, **25**, 402-408.
7. Dunn, K.W., Kamocka, M.M. and McDonald, J.H. (2011) A practical guide to evaluating colocalization in biological microscopy. *American journal of physiology. Cell physiology*, **300**, C723-742.

Supplementary Figure 1

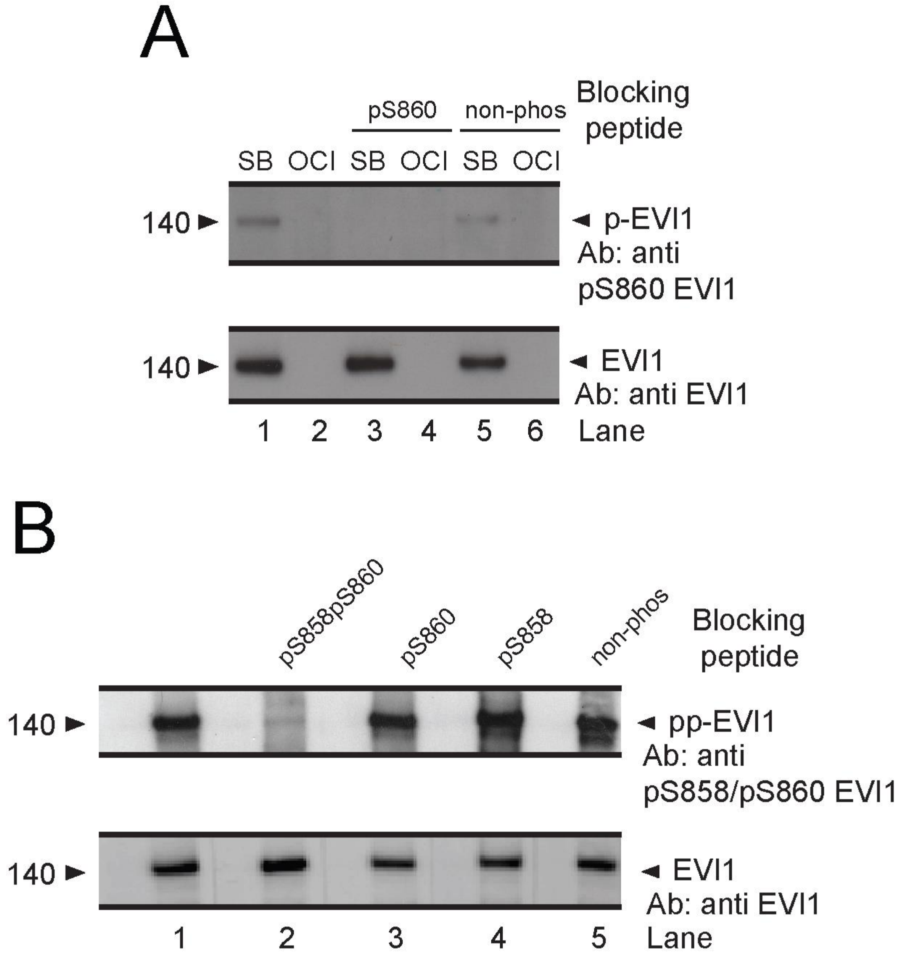

Supplementary Figure 2

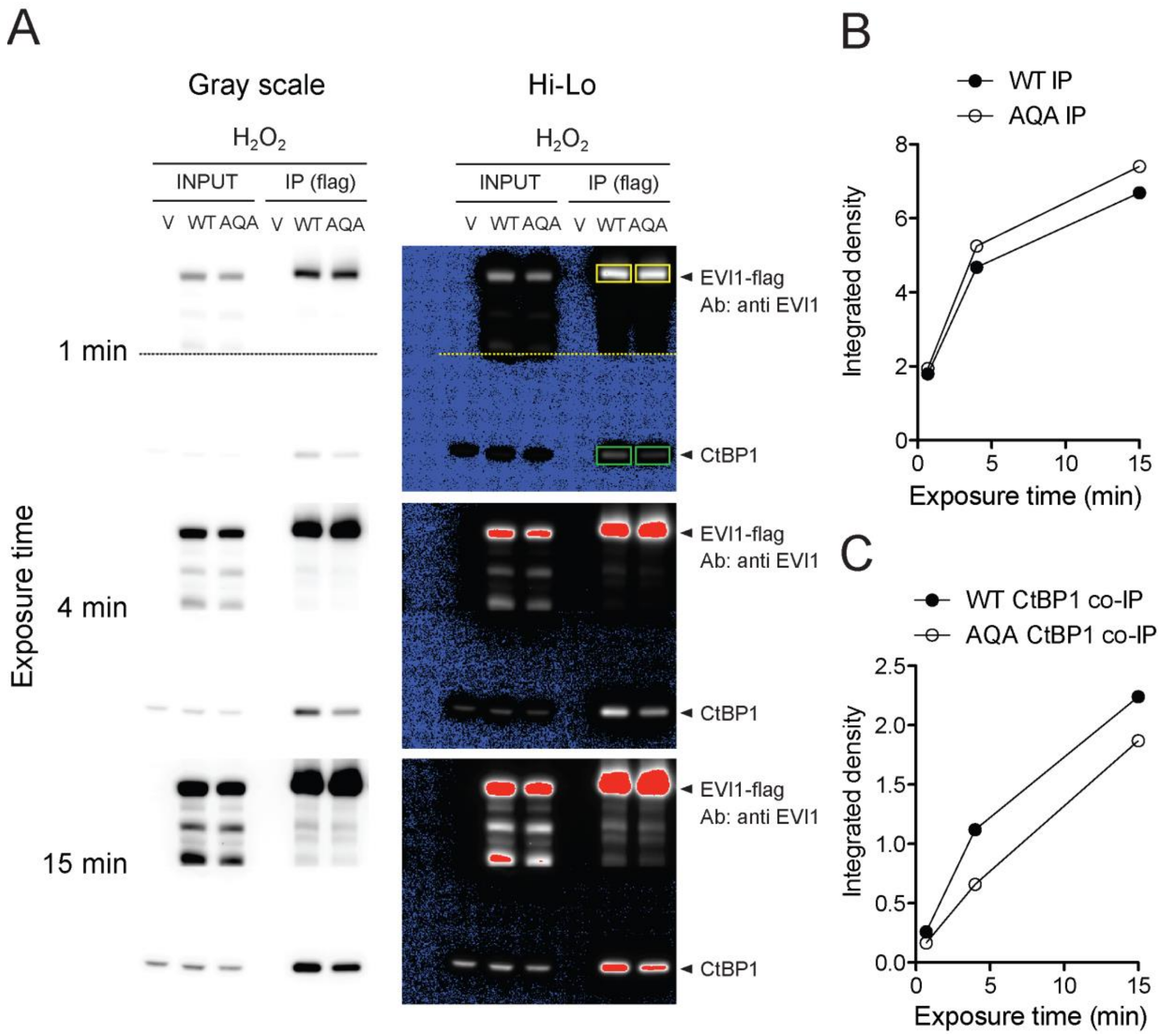

Supplementary Figure 3

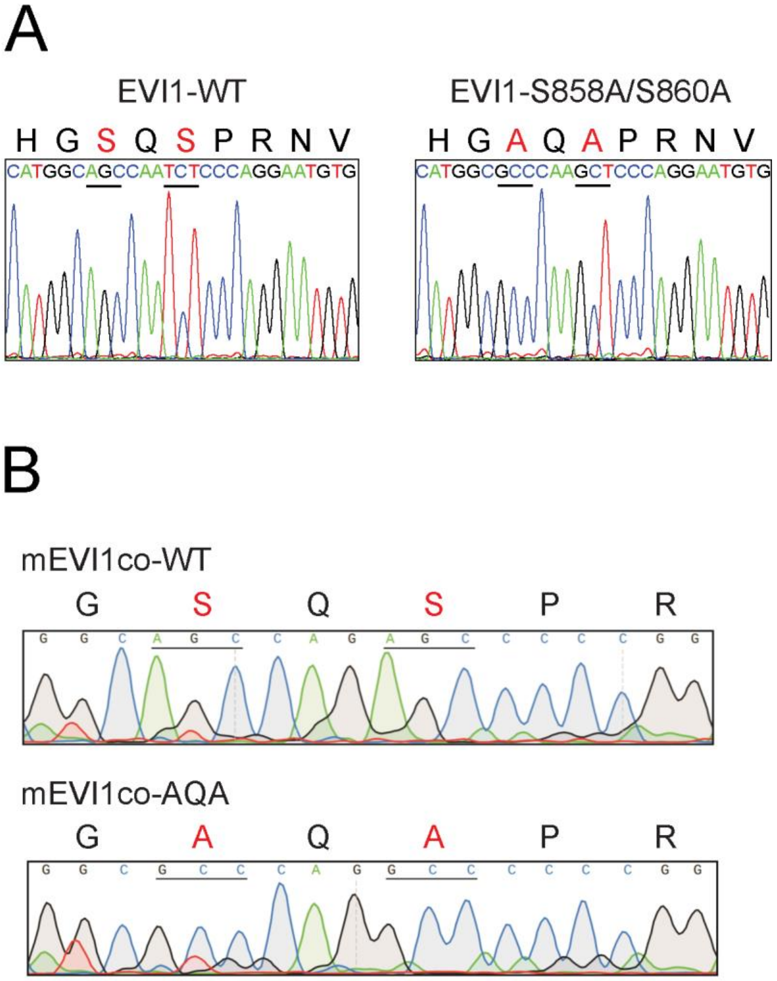

Supplementary Figure 4

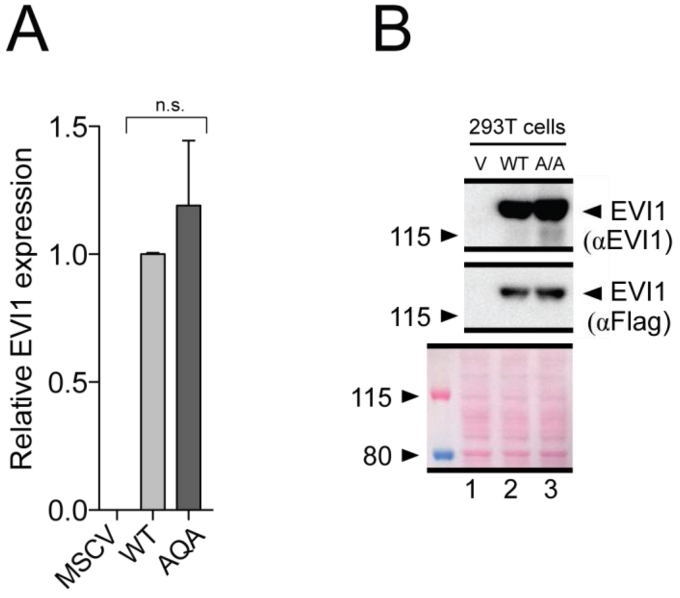

Supplementary Figure 5

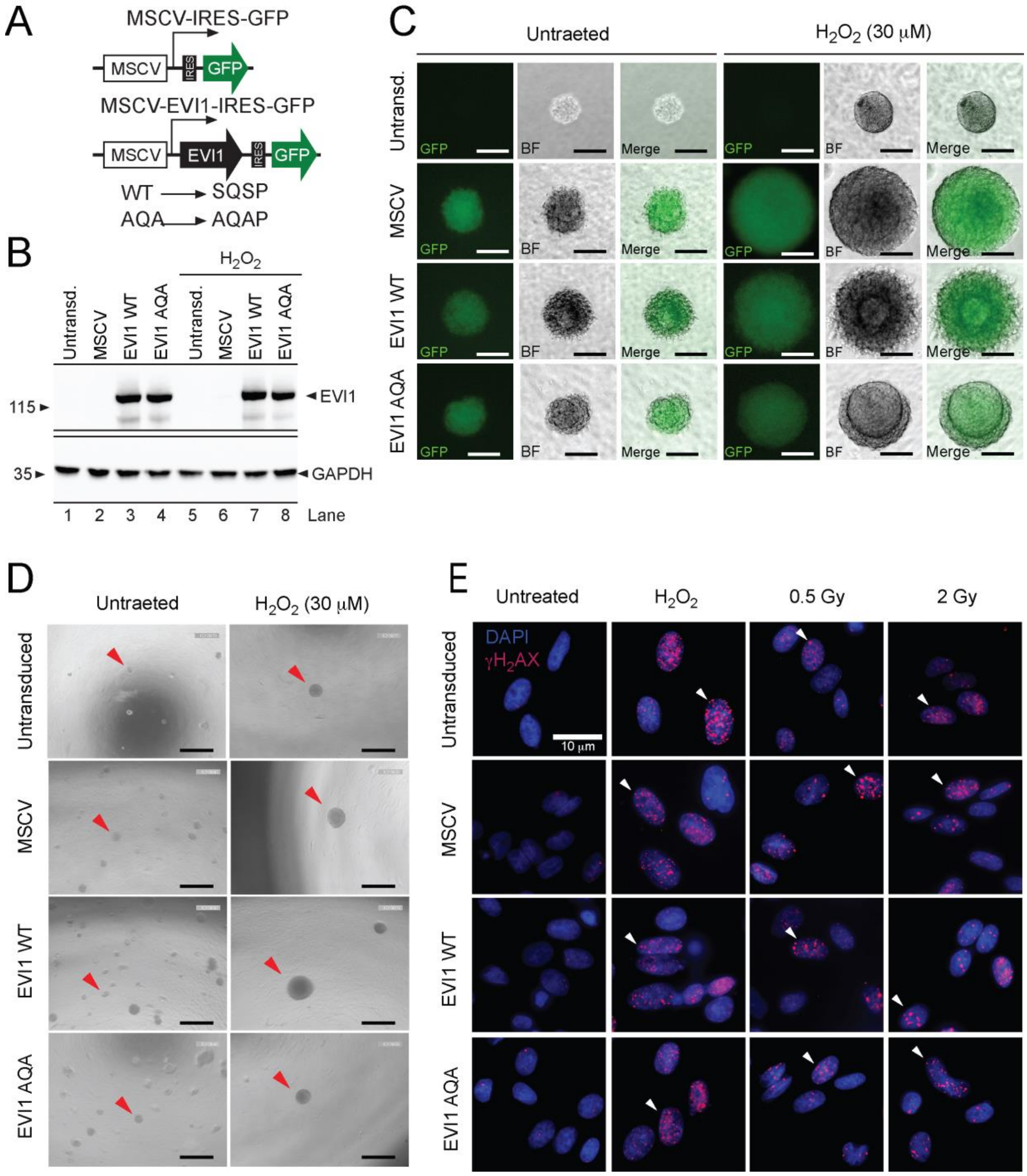

Supplementary Figure 6

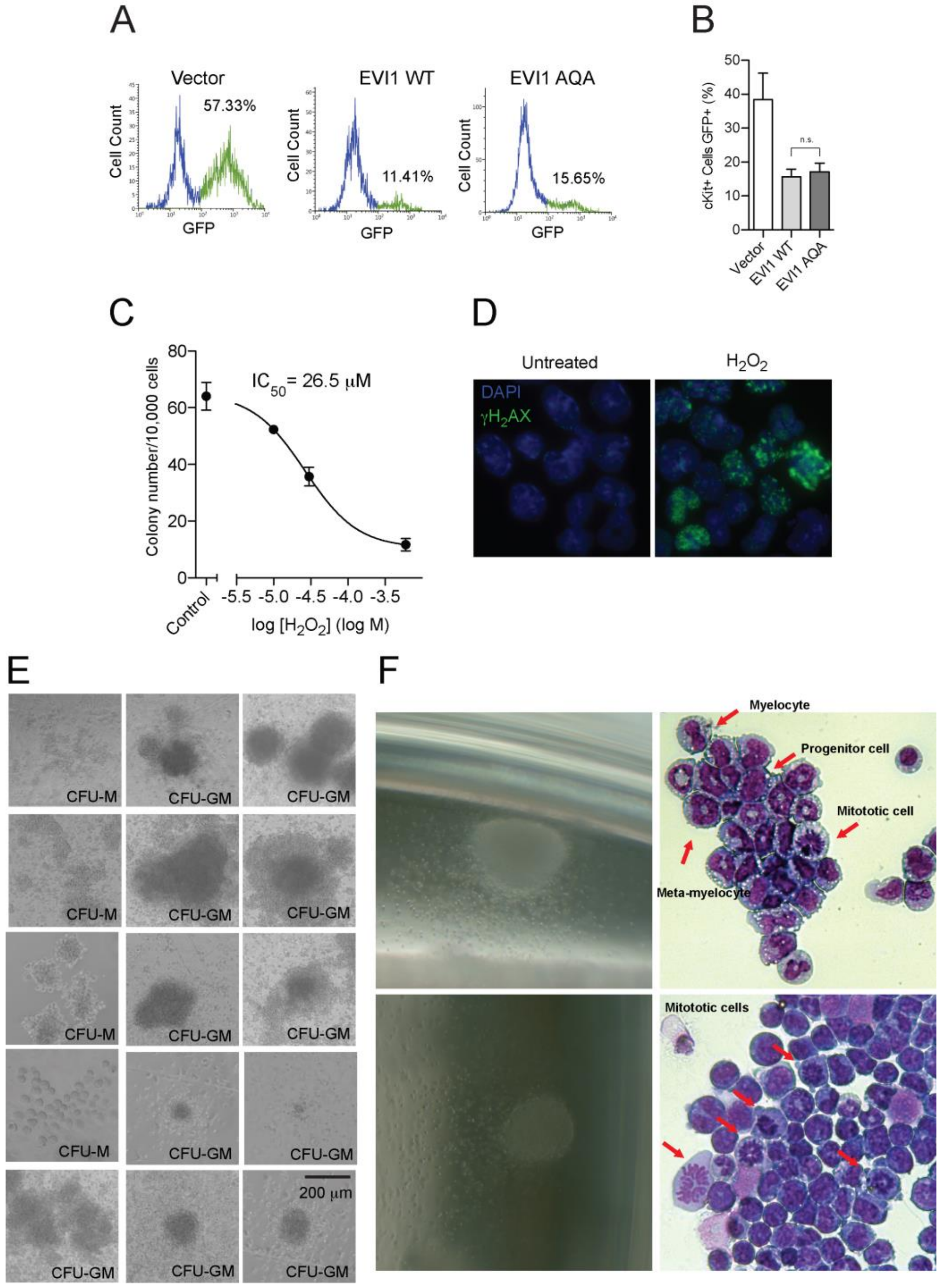

Supplementary Figure 7

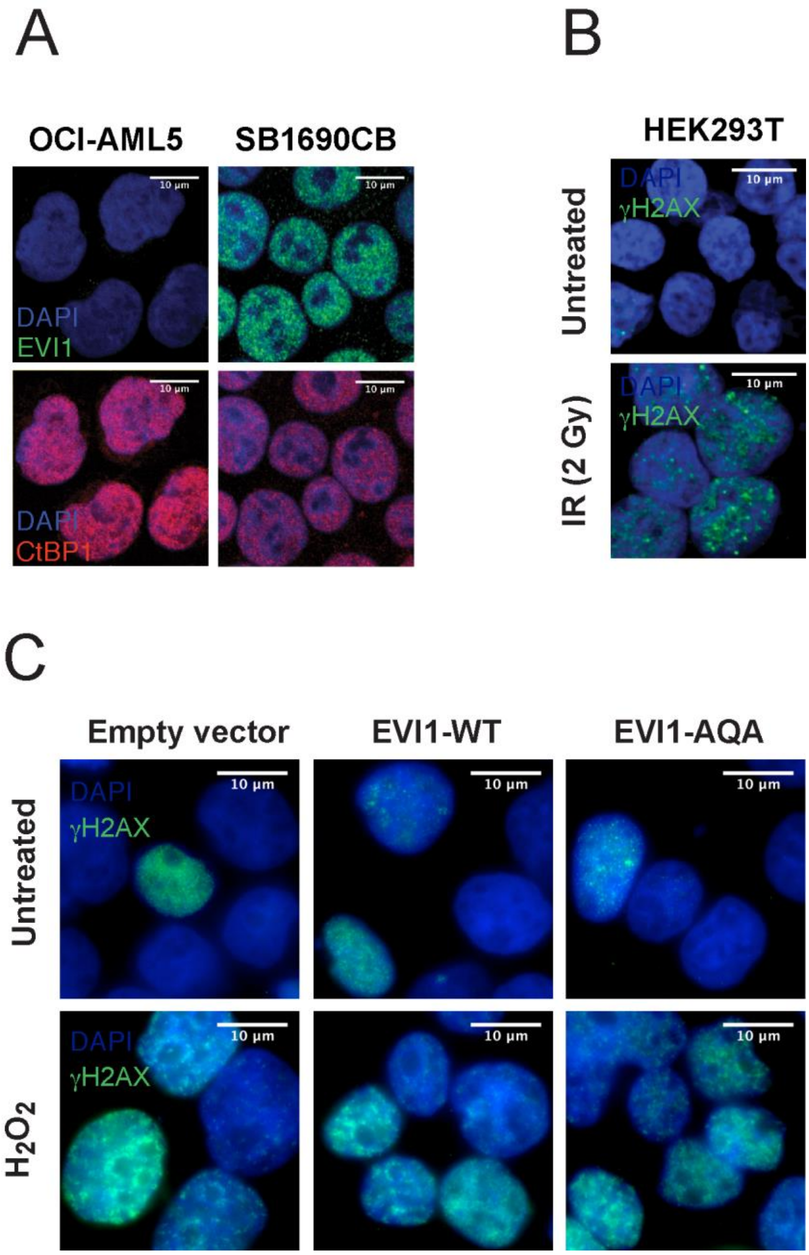

Supplement: Supplementary Data [file gky536_supplemental_files.zip › Suppl Material Paredes et al NAR-00443-V-2018 incl Figs Rev 1 .pdf]
